# Outcome measures in forensic mental health services: A systematic review of instruments and qualitative evidence synthesis

**DOI:** 10.1192/j.eurpsy.2021.32

**Published:** 2021-05-28

**Authors:** Howard Ryland, Jonathan Cook, Denis Yukhnenko, Raymond Fitzpatrick, Seena Fazel

**Affiliations:** 1 Department of Psychiatry, University of Oxford, Oxford, United Kingdom; 2 Nuffield Department of Orthopaedics, Rheumatology and Musculoskeletal Sciences, University of Oxford, Oxford, United Kingdom; 3 Nuffield Department of Population Health, University of Oxford, Oxford, United Kingdom

**Keywords:** Forensic mental health services, outcome measurement, psychometrics, quality of life, risk assessment

## Abstract

**Background:**

Outcome measurement in forensic mental health services can support service improvement, research, and patient progress evaluation. This systematic review aims to identify instruments available for use as outcome measures in this field and assess the evidence for the most common instruments, specific to the forensic context, which cover multiple outcome domains.

**Methods:**

Studies were identified by searching seven online databases. Additional searches were then performed for 10 selected instruments to identify additional information on their psychometric properties. Instrument manuals and gray literature was reviewed for information about instrument development and content validity. The quality of evidence for psychometric properties was summarized for each instrument based on the COnsensus-based Standards for health Measurement INstruments (COSMIN) approach.

**Results:**

A total of 435 different instruments or variants were identified. Psychometric information on the 10 selected instruments was extracted from 103 studies. All 10 instruments had a clinician reported component with only two having patient reported scales. Half of the instruments were primarily focused on risk. No instrument demonstrated adequate psychometric properties in all eight COSMIN categories assessed. Only one instrument, the Camberwell Assessment of Need: Forensic Version, had adequate evidence for its development and content validity. The most evidence was for construct validity, while none was identified for construct stability between groups.

**Conclusions:**

Despite the large number of instruments potentially available, evidence for their use as outcome measures in forensic mental health services is limited. Future research and instrument development should involve patients and carers to ensure adequate content validity.

## Introduction

Forensic mental health services provide care for people with mental illness who pose a risk to others and have typically perpetrated acts of violence or other antisocial behaviors [1]. The structure and legal framework governing such services varies considerably between and even within countries [2,3]. Demand for such services is rising in many high income countries, with increasing inpatient capacity [4]. Long length of stay, high staffing ratios, and the need for complex security arrangements mean that such services are expensive [5,6]. Forensic mental health services can consume a disproportionate portion of overall health budgets given the small numbers of patients [7]. Patients frequently spend many years in secure settings and continue to be subject to restrictions on discharge [8]. The consequences of recidivism are often severe for victims and their families [9]. Despite the financial and human costs, outcomes of care remain poorly understood and measurement of progress often relies on the individual approach of clinicians [10].

Measuring the outcomes of forensic mental health services is complicated. Unlike most other healthcare services which focus exclusively on improving outcomes for patients, forensic mental health services also have the dual purpose of public protection. In many jurisdictions this is considered their primary, if not sole, purpose. In other forensic mental health systems however, there is an increasing recognition that patient-centered outcomes must also be prioritized [11,12]. Previous research has frequently focused on objective outcomes, such as rehospitalization, reoffending and death, usually obtained from administrative datasets [13]. While such outcomes are clearly important, they are relatively uncommon and may only occur after considerable time has elapsed, limiting their usefulness to regularly monitor progress. Over the past three decades, there has been increasing interest in standardized questionnaires to quantify progress in a more nuanced way [14–16]. These questionnaires have predominately sought to reflect the assessment of the treating clinical teams, although more recent developments have also considered the views of patients themselves [17,18]. In practice, what constitutes progress varies considerably between services. Progress may be formally defined and based on objective criteria, for example as a move to a lower level of security or discharge to the community [19]. Alternatively, progress may be shown by more internal, less externally measured changes, such as a psychological shift toward responsibility for previous injurious actions [20]. Progress should therefore address therapeutic as well as risk reduction interventions. The questionnaires used to assess progress in clinical practice have not always been explicitly developed for this purpose [21]. Thus, dynamic risk assessment, needs assessments, and decision aids for determining the level of security have all been used as measures of outcome in forensic mental health services.

Policy programs are increasingly concerned with measuring outcomes across health services [22,23]. Driving principles highlight the need for measures to reflect the concerns of stakeholders, with adequate psychometric properties for their use as outcome measures [24]. The COnsensus-based Standards for health Measurement INstruments (COSMIN) group has developed a taxonomy to define the various qualities of an instrument that can make it a good outcome measure [25]. This includes aspects of validity, reliability, and responsiveness. Validity concerns whether an instrument actually measure the concept of interest, reliability whether it does so consistently, and responsiveness whether it is able to detect change over time. Instruments need to demonstrate good psychometric properties relevant to how they are used in practice. Measurement can be used at an individual level in determining a patient’s pathway. This can support patients to understand their own progress and to evaluate aspects of their treatment. It can also be used at a systemic level for quality assurance, allocation of resources, service evaluation, and research [26]. International initiatives have agreed common sets of outcome measures for similar clinical services and to be used in clinical trials to facilitate synthesis of individual study findings [27]. Understanding of psychometrics has evolved, placing greater emphasis on good content validity. Content validity asks the question of whether an instrument measures the concept that it is intended to measure. This concept should reflect those outcomes that are most important for stakeholders, including patients [28].

Previous reviews of outcome measures in forensic settings have identified a large number of questionnaire-based instruments in clinical practice and research settings [16,29]. These previous reviews noted a focus on risk and clinical symptoms, neglecting quality of life, and functional outcomes. They also highlight the lack of patient involvement in the development and rating of these instruments.

The present study seeks to update the evidence base, as previous reviews were completed almost a decade ago or only consider a small subset of measures [30]. It aims to identify existing instruments from published literature which have been, or could be used, as outcome measures. To ensure that the full range of instruments used in practice is included, we used a wide definition of what constitutes an outcome measure. This includes all instruments with a dynamic component that could be used to measure change over time, regardless of whether these were originally designed to be, or are termed as, an “outcome measure.” In this context dynamic components measured indicators that vary with time, where this variation may have a significant effect on the measurement result, in contrast to static items that measure historical factors, such as previous behaviors, which will not change on repeated measurement. Observed changes could be the result of a number of factors, including response to treatment and variations in symptoms. We decided to focus our quality assessment on instruments that are multidimensional, as these are more likely to be relevant to routine clinical practice in forensic services, where multiple outcomes are assessed for each patient. Although it is possible to combine many different instruments that are narrowly focused on measuring single domains, this can be cumbersome and time-consuming in clinical practice, and multidimensional instruments can reduce clinician burden. We gave equal weight to patient centered and service outcomes and the four outcome dimensions we consider are risk, clinical symptoms, recovery (including functioning), and quality of life. We also prioritize instruments that are specific to the forensic context, over more generic instruments. We identify the 10 instruments most frequently occurring within the literature that are also multidimensional and forensic specific. We then assess their quality, including development and content validity, drawing on the latest consensus-based approaches for evaluating instruments for the purpose of measuring outcomes from COSMIN [31]. To the best of our knowledge, this is the first review of this type to apply the COSMIN criteria to outcome measures in forensic mental health services. The purpose of the quality assessment was to determine how well the selected instruments function as outcome measures, using the COSMIN criteria as a benchmark, and not to determine the appropriateness of other potential uses for the included instruments, such as risk prediction or needs assessment.

## Methods

We report this review following the Preferred Reporting Items for Systematic Reviews and Meta-Analyses (PRISMA) reporting items, adapting where appropriate for this type of study [32]. We followed an adapted version of the COSMIN protocol for systematic reviews, including their risk of bias tool for assessing study quality [31].The COSMIN approach is an internationally agreed standard for evaluating outcome measures. It can be used to assess all types of outcome measures, including both clinician and patient reported instruments [33]. The study protocol was registered on PROSPERO, an international prospective register of systematic reviews.

### Step 1: Database search

We searched seven databases (MEDLINE, PsycINFO, CINAHL [Cumulative Index to Nursing and Allied Health Literature], EMBASE, National Criminal Justice Reference Service [NCJRS], the Cochrane Database, and Web of Science) from database inception until spring 2018 using a combination of terms including “tool”; “instrument”; “scale”; “outcome”; “recovery”; “risk”; “rehabilitation”; “quality of life”; “symptom”; “forensic”; “secure”; “unit”; “ward”; and “hospital”. See Supplementary Material 1 for an example of the full search strategy.

### Step 2: Screening and eligibility criteria

We reviewed the titles and abstracts of identified records. Included papers needed to describe the use of relevant instruments in a forensic mental health setting. The full text had to be available in English. All types of empirical or review paper were included. Papers describing use in prison or general psychiatric services only were excluded. Papers describing assessments of personality, which are generally not dynamic, and competency to stand trial and malingering, which are outcomes related to the legal process, rather than treatment response, were also excluded.

### Step 3: Full text review and identification of instruments

Papers meeting the screening criteria were reviewed in full text to identify relevant instruments described within. The format, type of study and geographical location were recorded. The frequency each instrument or subvariant appeared was noted. To determine the 10 most frequently appearing instruments, counts for all subvariants of each instrument were summed. We then considered each instrument, starting with the most frequently identified, to determine which met the criteria of being both multidimensional and designed for use in a forensic mental health context, until we had identified the 10 most frequently occurring within the literature. Multidimensional instruments included items on more than one of the four domains identified in previous reviews in this field (clinical symptoms, risk, recovery, and quality of life) [15,16]. Forensic specific instruments were those concerned with mental health outcomes for offenders or outcomes for individuals assessed or treated in forensic mental health services. We then considered each of the 10 selected instruments to determine the most relevant version or variant to undergo quality assessment in the next stage of the review. This was either the most recent version or, for instruments that combined multiple components, those components designed to measure patient progress over time.

### Step 4: Further searching for literature on selected instruments

We conducted additional searches for each of the 10 selected instruments. We searched the PubMed database using common variants of each instrument’s name combined with the COSMIN filter of psychometric terms [34]. We reviewed the manuals for each instrument and other gray literature for further information on instrument development. We reviewed the reference lists of all included papers and contacted experts in the field as necessary. All sources of information were included until the end of 2019.

### Step 5: Data extraction

We developed a data extraction tool, based on the COSMIN systematic review protocol and risk of bias tool. A number of adaptations to the standard approach were necessary, as the identified instruments were predominantly clinician reported. Content validity focused on the qualitative comprehensiveness and relevance of items in relation to the concept of interest, while all other psychometric properties were assessed using quantitative studies of numerical scores generated by the instruments. Quantitative data were extracted on seven psychometric properties (structural validity, internal consistency, measurement invariance, reliability, measurement error, hypothesis testing, and responsiveness). According to COSMIN, the dimensionality of a scale should be determined by factor analysis before internal consistency is considered [35]. In this context, dimensionality considers whether there is statistical evidence that respondents answer an instrument’s items in a similar way, indicating that they relate to the same underlying construct. Measurement error refers to the systematic or random error of a patient’s score that is not attributable to true changes that have occurred. It requires a qualitative estimation of the minimal important change, which is the smallest change in a score that would be clinically meaningful. We assigned a quality rating to the evidence for each property for each instrument in each study in one of three categories (no concerns/quality of evidence unclear/quality of evidence inadequate).

### Step 6: Overall strength of evidence

We assigned an overall rating to the strength of evidence available for each of the seven psychometric properties for each instrument based on all included studies in one of three categories. For properties with adequate evidence of good performance we assigned the highest category. Properties with either inadequate evidence of good measurement properties or evidence of inadequate measurement properties were assigned to the middle category. Those properties with no evidence were assigned to the lowest category.

We used the same categorization system for content validity, including the instrument development process. However, due to the lack of published studies, we used a qualitative synthesis of information available from a range of sources, including instrument manuals and other gray literature, based on the COSMIN methodology for assessing content validity, which focuses on establishing relevance and comprehensiveness in the target population [36].

## Results

### Description of full text articles retrieved

The initial screening process identified 4,494 unique references, of which 502 met the inclusion criteria for full text review. Four hundred and fifty-six (91%) were articles in scientific journals. Almost half (49%; *n* = 247) were studies of the psychometric properties of instruments, while only 3% (*n* = 17) concerned interventional trials. Almost half (45%; *n* = 227) originated in the UK and Ireland (see Supplementary for a full description of the studies reviewed in full text).

### Description of the instruments identified

Four hundred and thirty-five different instruments or their variants of were identified. It was necessary to review 14 instruments until we identified the 10th instrument most frequently occurring within the literature that also met the multidimensional and forensic-specific criteria (see Supplementary Material 3). The most frequently occurring instrument within the literature was the Historical, Clinical, Risk 20 (HCR-20) [37], which appeared 196 times, followed by the Short Term Assessment of Risk and Treatability (START) [38] with 53 mentions.

There was considerable variation in the format and stated purpose of the selected instruments. This included assessments of progress, risk factors, protective factors, patient need, and clinical decision aids.

### Overview of the 10 instruments selected for the quality assessment

Half of the 10 instruments assessed were developed primarily as risk assessments (HCR-20, START, Sexual Violence Risk 20 [SVR-20], Violence Risk Scale [VRS], Level of Service: Case Management Inventory [LS/CMI]) [37–42]. Two instruments explicitly included items on patients’ strengths or protective factors (START and SAPROF) [38,43]. Only one instrument, the Health of the Nation Outcome Scale Secure (HoNOS Secure), was explicitly developed as a progress measure [44]. All instruments included a clinician reported scale. Only one, the Camberwell Assessment of Need Forensic Version (CANFOR), was originally developed to include a patient reported scale [18]. A patient reported scale has subsequently been developed for the Dangerousness, Understanding, Recovery, and Urgency Manual (DUNDRUM) [17]. The number of items ranged from 12 (DUNDRUM 3 and 4) to 150 Behavioral Status Index (BEST) [45,46]. See Table 1 for a full description of each of the instruments.

**Table 1:** An overview of the 10 outcome measurement instruments included in the quality assessment

**Measurement Instrument** **(Key reference)**	Construct	Target population	Mode of administration	Recall period	Subscale and number of items	Response options	Ranges of scores for individual items	Original language
Historical Clinical Risk 20 (HCR-20) Version 3 [37]	Static and dynamic risk factors for violence	Correctional, civil psychiatric and forensic psychiatric settings	Clinician reported	Lifetime for historical scale, timeframe for clinical and risk scales determined for each patient by raters	20 items in 3 subscales:	Presence - yes, partially or possibly present, no, omit	0-2	English
					Historical (10 items), Clinical (5 items), Risk (5 items)	Relevance – high, moderate, low, omit		
						Structured professional judgement for future violence, serious physical harm and imminent violence can be high, moderate or low	High/mod/low	
Short-Term Assessment of Risk and Treatability (START) [38]	Strengths and vulnerabilities	Forensic mental health patients	Clinician reported	2-3 months (or since the last START assessment)	40 items in 2 parallel subscales, plus 2 case specific items	None, low, high	0-2	English
					Strengths and vulnerabilities (20 items each, plus 2 case specific items)	Strengths can be marked as ‘key items’ and vulnerabilities as ‘critical items’	Yes/no	
					Specific risk estimates (7 SREs)	SREs can be high, moderate or low	High/mod/low	
Camberwell Assessment of Need – Forensic Version (CANFOR) [18]	Assessment of needs	Forensic mental health patients	Clinician and patient reported scales	1 month	25 items in 1 scale	No problem/moderate problem/serious problem/not known OR None/low help/moderate help/high help/not known	Variable: 0-2 or 0-3	English
							Aggregate scores of met needs, unmet needs and total needs	
Dangerousness, Understanding, Recovery and Urgency Manual (DUNDRUM) [45]	Readiness to move to a lower level of security	Forensic mental health patients	Clinician and patient reported scales	Variable – 5 years for score 0, unclear for the other scores	12 items in 2 subscales:	Ordinal: A statement corresponds to each of five possible scores	0-4	English
					DUNDRUM3 - Programme Completion (7 items);			
					DUNDRUM4 - Recovery (5 items)			
Health of the Nation Outcome Scales – Secure Version (HoNOS Secure) [44]	Repeatable progress measure for forensic services	Forensic mental health patients	Clinician reported (any mental health professional)	The HoNOS-Secure Clinical/social functioning scale – previous 2 weeks Security scale – the ‘near future’	19 items in 2 subscales:	Ordinal: Examples of each rating point provided in the glossary	0-4	English
					Clinical/social functioning (12 items); Security (7 items)			
Level of Service: Case Management Inventory (LS/CMI) [42]	Risk factors for recidivism, intervention needs and case management	Offenders in a variety of settings, including prison, psychiatric hospitals and probation	Professional reported	Variable, depending on the item – where specified, usually the last year	43 items in Section 1 - General risk/need in 8 subscales – Criminal History (8), Education/Employment (9), Family/Marital (4), Leisure/Recreation (2), Companions (4), Alcohol/Drug Problem (9), Procriminal Attitude/Orientation (4), Antisocial Pattern (4)	Ordinal or binary	3-0 or Yes/No	English
					4 additional scales that do not add to the score, but are considered in administrative override and/or case management: Specific risk/need (21), prison experience/ institutional factors (11), other client issues (21), special responsivity considerations (11)	Final risk/need assessment	Very high, high, medium, low, very low	
Violence Risk Scale (VRS) [41]	Risk factors for violence, readiness for change, targets for intervention, effect of treatment	Forensic inpatients and prisoners	Clinician reported – file review and semi-structured interview	Lifetime functioning, with emphasis on recent functioning	26 items in 2 subscales:	Ordinal: Responses depend on the item	0-3	English
					Static (6) Dynamic (20)			
Structured Assessment of Protective Factors for risk of violence (SAPROF) [43]	Protective factors for violence	Forensic psychiatric inpatient and outpatients; prisoners and probation	Clinician reported	Information used from the last 6 months; predictions apply to subsequent 6 months	17 items in 3 subscales:	Each item is rated on a 3-point scale	0, 1 or 2	Dutch
					Internal (5) Motivation (7) External (5)	Final protection judgement: 1) Protection 2) Risk	High/mod/low	
Sexual Violence Risk 20 (SVR-20) [40]	Risk factors for sexual violence	Sex offenders (including those who are forensic psychiatric patients)	Clinician reported	Recent changes within the last year (can be adjusted to each case)	20 items in 3 subscales:	Presence - yes, partially or possibly present, no, omit	0-2	English
					Psychosocial adjustment (11); sexual offences (7); future plans (2)	Recent change	+, 0, -	
						Summary risk rating	High/mod/low	
Behavioural Status Index (BEST) [46]	Assessment of behaviours	Forensic and general psychiatric inpatients	Nurse reported	Last 3 months	150 items in 6 subscales:	Ordinal: Responses depend on the item	1-5	English
					Social Risk (20); Insight Subscale (20); Communication and Social Skills (30); Work and Recreational Activities (20); Self-Care and Family Care (30); Empathy (30).			

### Quality of evidence for the selected instruments

**Figure 1.**
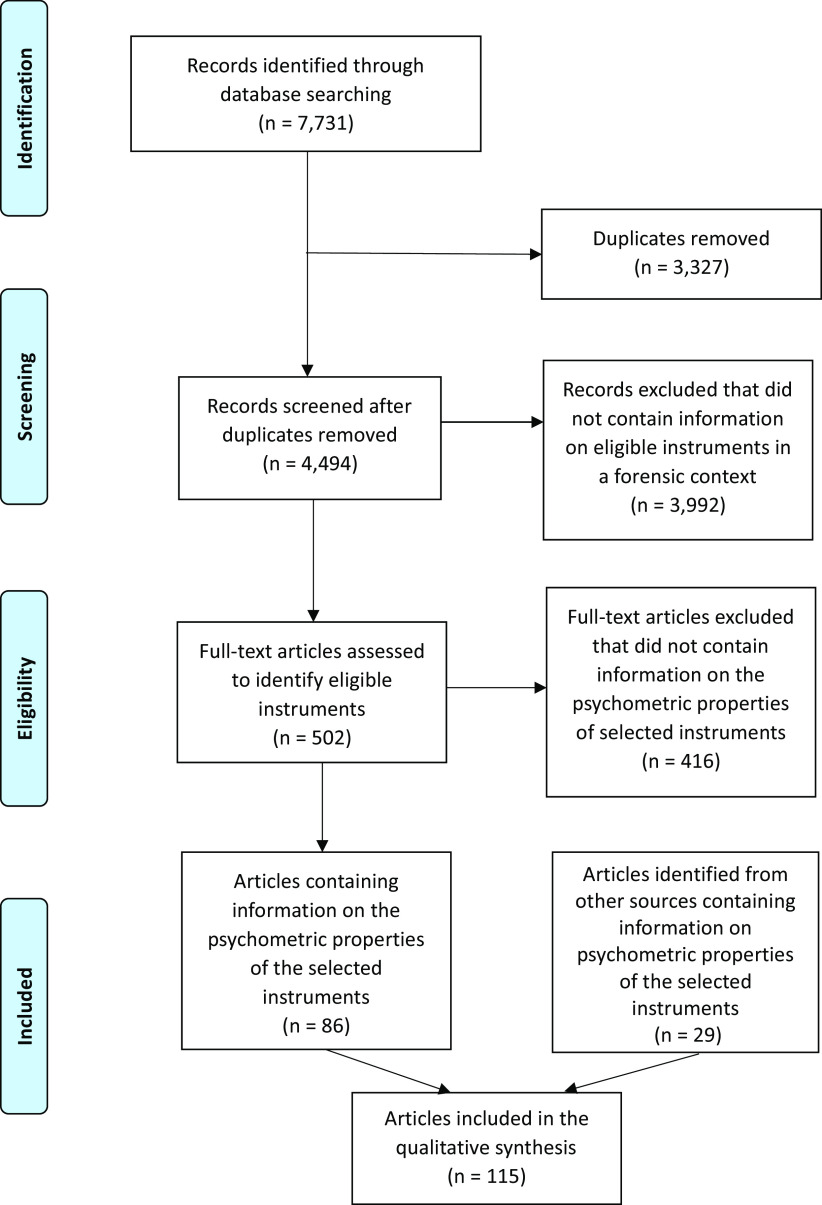
PRISMA flow diagram showing the flow of studies through the review.

Eighty-six (17%) of the references identified by the review strategy contained relevant data on the psychometric properties of the 10 selected instruments. An extra 29 references were identified through the additional search techniques described in Step 4 of the methods (see Figure 1). See Supplementary Material 4 for details of the identified studies containing psychometric information about the selected instruments.

All 10 selected instruments had some evidence of empirical processes to support their development, however, this often emphasized quantitative reviews of the literature on risk factors for violence, rather than considering the views of relevant stakeholders [36]. When there was evidence of consultation with stakeholders, this was usually unstructured, with limited details on the methods used or individuals involved. Only one instrument, CANFOR, demonstrated adequate evidence of stakeholder involvement, including patients, in its development [18]. CANFOR also had evidence to support its relevance and comprehensiveness for the target population.

The degree of evidence for the remaining psychometric properties was mixed, with evidence on testing hypotheses for construct validity identified for every instrument, but none for measurement invariance [35]. Evidence for structural validity was available for three of the instruments (BEST, VRS, and DUNDRUM), none of which demonstrated adequate performance [41,45,47]. This was either due to insufficient numbers, the use of exploratory, rather than confirmatory factor analysis, or results that were not supportive of the hypothesized structure of the instrument [31]. There was evidence for internal consistency identified for 8 instruments out of 10. Despite the lack of evidence for structural validity, four instruments were deemed to have evidence of adequate internal consistency.

Nine instruments had some evidence for their reliability, which focused primarily on interrater, rather than test–retest reliability. Measurement error had limited evidence with studies identified for three instruments [48–50]. The quality of evidence for measurement error in the review was consistently low, relying on quantitative methods alone, with no attempt to relate the statistical error to the minimal important clinical change [31]. Testing hypotheses for construct validity was the category with the greatest quantity of evidence. Three primary types of hypotheses were identified: prediction of future events, such as violence, self-harm, and victimization; difference between subgroups, based on characteristics such as sex, ward type or behavior; and correlation with other measures. Evidence for responsiveness was identified for seven instruments, with only two demonstrating adequate properties in this respect [48,51]. See Table 2 for an overview of the evidence for the selected instruments and Supplementary Material 5 for a detailed summary.

**Table 2.** Summary synthesis of evidence for the 10 outcome measurement instruments included in the quality assessment.

	Content validity	Structural validity	Internal consistency	Measurement invariance	Reliability	Measurement error	Hypothesis testing for construct validity	Responsiveness
HCR-20		0	4	0	10	0	17	3
START		0	5	0	10	0	28	2
CANFOR		0	0	0	4	0	10	0
DUNDRUM		1	4	0	1	1	7	1
HONOS-S		0	2	0	1	1	14	11
LS/CMI		0	1	0	0	0	3	0
VRS		1	1	0	7	4	11	8
SAPROF		0	2	0	8	0	12	2
SVR-20		0	0	0	2	0	5	0
BEST		2	3	0	3	0	4	2

*Note*: This table provides an overall summary of the evidence for the psychometric properties of each of the included measurement instruments. The eight psychometric properties assessed are listed at the top of the table and the 10 instruments on the left hand side. The numbers in the cells signify the number of studies identified which contain information about the relevant psychometric property for each instrument. Numbers are not included for content validity, as this was not possible to accurately quantify, due to the diverse range of sources of information for this property. The shading categorizes the level of evidence within each cell according to the schedule outlined below: Adequate evidence of good measurement properties Inadequate evidence of good measurement properties or evidence of inadequate measurement properties No evidence

Definition of terms used in Table 2.

**Table:** 

Term	Definition
Content validity	The degree to which the content of an outcome measure is an adequate reflection of the construct to be measured
Structural validity	The degree to which the scores of an outcome measure are an adequate reflection of the dimensionality of the construct to be measured
Internal consistency	The degree of the interrelatedness among the items
Measurement invariance	The degree to which respondents from different groups with the same latent trait level respond similarly to a particular item
Reliability	The extent to which scores for patients who have not changed are the same for repeated measurement under several conditions: for example, over time (test–retest) or by different persons on the same occasion (inter‐rater)
Measurement error	The systematic and random error of a patient’s score that is not attributed to true changes in the construct to be measured
Hypothesis testing for construct validity	The degree to which the scores of an outcome measure are consistent with hypotheses based on the assumption that the outcome measure validly measures the construct to be measured
Responsiveness	The ability of an outcome measure to detect change over time in the construct to be measured

## Discussion

This systematic review aimed to provide an overview of instruments currently available for use as outcome measures in forensic mental health services. A broad definition of what constitutes an outcome measure ensured a wide range of instruments were considered. The review focused on instruments which are clinically relevant, to increase applicability of findings to real world settings. It assesses the quality of evidence for the 10 most frequently occurring instruments within the literature, which are also multidimensional and forensic specific. This review was based on a recognized quality assessment process and, to our knowledge, this is the first time that such a systematic approach has been applied in this field [35]. This quality assessment specifically considered the use of these instruments as broad outcome measures, covering a wide range of clinically relevant domains. It made no evaluation about the use of instruments for other purposes, such as risk prediction or needs assessment.

### Key findings

Overall, the evidence for the appropriateness of the selected instruments as broad outcome measures is limited (see Table 2). At least half focused primarily on risk assessment and management, which is in line with previous similar reviews and unsurprising given the nature of forensic mental health services [15–18]. The Overt Aggression Scale, developed to measure aggressive behavior in inpatients with intellectual disabilities, appeared frequently but was excluded from more detailed assessment due to not being multidimensional [52]. Although clinical symptoms of mental illness featured in many of the selected instruments, this was not the primary focus of any. The Positive and Negative Symptoms Scale [53] and Brief Psychiatric Ratings Scale [54] both appeared frequently, but were excluded from more detailed assessment as they only focused on symptoms and were not designed for use in a forensic context (see Supplementary Material 3). Recovery and quality of life were less prominent in the selected instruments, although there were both some generic and forensic specific measures of these domains in the other instruments identified (such as the Global Assessment of Functioning [55], which appeared frequently but was excluded as not forensic specific or multidimensional, or the forensic specific Lancashire Quality of Life Profile [56], which did not appear frequently enough to warrant more detailed assessment). In accordance with previous reviews, few instruments were reported by patients, with only 2 of the 10 selected instruments having a patient reported scale [15,16]. The systematic gathering of the views of a wider group of stakeholders, especially patients, was rarely performed to inform content validity.

The differing attention to various aspects of validity and reliability in the quality assessment reflects the original purposes of the instruments. For example, as an assessment of patient need, the CANFOR has a much greater focus on content validity, while the HCR-20, as a risk assessment, focuses more on prediction of negative outcomes [18,57]. Studies of the DUNDRUM quartet often focus on differences between levels of security, as an aid to support decisions on pathway placement rather than outcome measurement, while the HoNOS-Secure has the highest number of studies of responsiveness, commensurate with its role as a progress measure [45,58].

### Implications for research

The COSMIN guidelines emphasize the need for outcome measures to demonstrate adequate stakeholder involvement in their development [28]. Even for clinician reported scales, this should include input from patients and carers. This holds for forensic services, which must balance the needs of patients with those of public protection. Evidence for instrument development was only adequate for the CANFOR [18]. Although other selected instruments had some stakeholder involvement, this was limited, unstructured and the reporting often inadequate. Subsequent empirical validation of instrument content was similarly lacking, again except for CANFOR. Testing of comprehensiveness and relevance should be completed in the population for which instruments are intended [28]. This can take place after the instrument is available in its final form and does not have to occur contemporaneously with development [31]. Further research is therefore necessary to establish the content validity of these instruments as outcome measures in a forensic psychiatric population.

Overall, the evidence for the other psychometric properties of the instruments as outcome measures is limited, with numerous gaps in the published research. This lack of a comprehensive evidence base is perhaps surprising given the age and popularity of many of these instruments, but may reflect the diversity of their intended uses. Adequate evidence for other uses, such as risk predication, may be well established, but does not necessarily support their use as outcome measures. Further research should seek to ensure that the identified gaps in the evidence base are addressed, if these instruments are used as outcome measures. Certain properties, such as measurement error and measurement invariance are almost entirely overlooked, so should be considered in future studies. Evidence for other fundamental characteristics, such as structural validity, is also often absent or inadequate.

The ability to detect change over time was explicitly considered in the review under the category of responsiveness. While seven instruments had some evidence for responsiveness, this property was only deemed adequate for the VRS and SAPROF. Demonstrating reliable change in this population can be challenging, due to the long timescales involved [59]. Admissions to inpatient forensic psychiatric care often last years. The timeframe of most psychometric studies however, including many in this review, is limited to a few months [8]. Despite these difficulties, it is essential for outcome measures to demonstrate responsiveness to change over a time period that is relevant for the population of interest [60].

Authorship bias has been identified as a potential problem in the literature on risk assessments in the forensic context [61]. While authorship bias was not formally assessed in this review, much of the evidence identified was produced by the teams that originally developed the instruments. Sufficient validation studies should therefore be conducted independently of the original authors.

New instruments are needed for forensic mental health services to enable clinicians and patients to report and measure individual and service outcomes. These should be developed according to the latest best practice guidelines, including the participation of relevant stakeholders, such as clinicians and patients [62,63]. Developing new instruments will require working with these stakeholders to identify and prioritize the most important outcomes. This should be followed by further work to develop an instrument that fits the needs of individuals and services. Finally, empirical studies should confirm adequate psychometric properties for the new instrument, such as content validity and responsiveness.

### Implications for policy and practice

This review identified many instruments that have been, or could be, used as outcome measures in forensic mental health services. These vary considerably in format, content, length, stated purpose, and evidence base. Of the 10 instruments reviewed in detail, only HoNOS-Secure is designed with the sole primary purpose of measuring progress, although other instruments such as the VRS and LS/CMI are also intended to assess change over time [44]. The ways that clinicians and researchers use instruments can differ considerably. Risk assessments, such as the HCR-20, can be used by clinicians to develop risk formulations, while researchers may use it to predict negative outcomes. Instruments can be used in practice or in research in several different ways, for example using the same instruments to predict the risk of future events and to establish if an intervention has already reduced that risk [64]. This type of repurposing may be possible, but is limited by how to interpret scores. It will also need considerable additional work to establish relevant psychometric properties, in particular adequate content validity and responsiveness [28,60]. While some commonly used instruments, such as HCR-20 and START, have been used as outcome measures, the underlying evidence for their use in this way is weak. Use of such risk assessments as outcome measures in isolation may lead to an unbalanced view of progress, as they do not include important outcomes such as quality of life and social functioning. Services should therefore start by deciding which outcomes are important, before selecting high quality outcome measures that cover all such outcomes in a way that is practical to implement.

Most instruments identified in this review are reported by clinicians only. For instruments that do include a patient reported scale, these scales may have been designed after the development of the clinician reported ones, with limited patient input [17,65]. This risks inadequate attention to the patient perspective in the overall design and implementation of such measures [66]. In instruments selected in this review that include a patient reported scale, the patient reported scales mirror their clinician reported components. They contain identical items, reframed from the patient’s perspective, to allow direct comparison between the two scales. A disadvantage of this approach is that certain outcome areas, such as those related to subjective quality of life, may only meaningfully be rated by patients [67]. A patient reported scale that exactly mirrors the clinician reported scale therefore risks neglecting such areas. Services wishing to implement patient reported measures should consult their own users and other key stakeholders, such as family members, when selecting scales, to ensure that they are fit for the purpose of measuring those outcomes deemed of greatest relevance [11].

Comprehensiveness is an essential quality for outcome measures [28]. While risk and clinical symptoms are the dominant domains within the most frequently occurring instruments within the literature, quality of life and functional outcomes are either absent or remain of secondary importance. By relying on existing instruments services may overlook outcomes of importance, such as quality of life, and over-emphasize the importance of other domains, such as risk to others [68].

### Limitations

Given the very large number of instruments identified, it was only possible to assess the quality of evidence for a small proportion of them. Some of the included instruments were not intended to be used as outcome measures, and their utility is not limited to this. A frequency based approach was chosen to select instruments for the quality assessment. This was deemed the most systematic method of identifying instruments that were likely to have a sufficient evidence base to judge their qualities against the COSMIN criteria. There may be instruments that did not meet our selection criteria that have the potential to perform well against the COSMIN criteria, when sufficient evidence is available. The use of frequency of appearance in the literature to select instruments for quality assessment has a number of drawbacks. Firstly, older tools are likely to appear in more published studies, simply by virtue of being in existence for longer. Secondly, some studies were published as multiple papers, meaning that a limited evidence base generates a disproportionate number of references. Thirdly, although we grouped variants of instruments together, there may be important differences between variants. Finally, all types of paper were included in the count and the proportion of studies that contained psychometric information on a particular instrument varied, so the overall count does not necessarily reflect the quantity of psychometric evidence available.

Language was a limitation in two ways. Firstly, the search was limited to those references where the full text was available in English. Secondly, studies involving translations of instruments were included, although evidence from a translated version may not always apply directly to the English version, due to subtle cultural and linguistic differences [69].

Assessing the quality of instrument development and content validity studies according to the full COSMIN criteria proved challenging [28,35]. The review team simplified the COSMIN approach, to make it more pragmatic and streamlined. This included reducing the quality assessment to three levels, rather than four. The summary assessment of the quality of evidence for instrument development and content validity was also simplified, as the limited evidence in this area rendered the full process recommended by COSMIN unworkable. Despite these limitations, we think that the COSMIN framework is the most robust and relevant mechanism currently available for assessing instruments for use as outcome measures.

### Conclusions

Although there are a large number of instruments available that can be used as outcome measures in forensic mental health services, the evidence base for their use in this way is limited. Despite recommendations from previous reviews, instruments that appear most frequently in the literature remain focused on risk and fail to adequately involve all stakeholders, especially patients [15,16]. Repurposing instruments developed for other uses as outcome measures should be avoided where possible. This is particularly the case for risk assessment tools which cannot currently be recommended as outcome measures based on the standard guidelines we have outlined. When this is unavoidable, additional research is necessary to ensure that they demonstrate adequate psychometric properties to be used as outcome measures [35]. New outcome measures should be designed with input from all relevant stakeholder groups, especially patients and carers, who have hitherto been largely ignored [67]. This should follow current best practice guidelines for outcome measure development, with a focus on ensuring adequate content validity [28].

## Data Availability

The data that support the findings of this study are available from the authors on reasonable request.

## Supplementary Materials

To view supplementary material for this article, please visit http://dx.doi.org/10.1192/j.eurpsy.2021.32. click here to view supplementary material
