# Supplementary material for: Outcome measures in forensic mental health services: A systematic review of instruments and qualitative evidence synthesis
Source: Eur Psychiatry. 2021 May 28;64(1):e37. doi: 10.1192/j.eurpsy.2021.32 (PMC8260563; doi:10.1192/j.eurpsy.2021.32)
Supplement: Supplementary file 1 [file S0924933821000328sup001.zip › S0924933821000328sup004.docx]

**Table A3**

*Studies included in the systematic review*

|  | **Population** | | | **Instrument administration** | | | | | **Measurement instrument** | | | | | | | | | |
| --- | --- | --- | --- | --- | --- | --- | --- | --- | --- | --- | --- | --- | --- | --- | --- | --- | --- | --- |
| **Reference** | **N** | **Age – mean (SD)** | **Sex**  **(% male)** | **Setting** | **Participants** | **Country** | **Language** | **Version(s)** | **HCR-20** | **START** | **CANFOR** | **DUNDRUM** | **HoNOS-S** | **LS/CMI** | **VRS** | **SAPROF** | **SVR-20** | **BEST** |
| (Abidin et al., 2013) | 98 (out of 100 eligible) | 40.45 (12.8) | 94 | High, medium and low security hospital | Forensic psychiatric inpatients | Ireland | English | SAPROF – Standard; DUNDRUM- ; START – Standard; HCR 20 – V3 | X | X |  | X |  |  |  | X |  |  |
| (Abou-Sinna & Luebbers, 2012) | 72 | 37.8 (8.98) | 92 | Forensic psychiatric hospital | Forensic psychiatric inpatients | Australia | English | LS/CMI -General needs only; HoNOS-Secure – Version 2 |  |  |  |  | X | X |  |  |  |  |
| (Adams et al., 2018) | 327 (25 in prison) | Male 45.2; female 42.9 | 90 | Forensic psychiatric hospitals (HSU, MSU, LSU, open security); community forensic psychiatric teams; prison | Forensic psychiatric inpatient and outpatients; prisoners | Australia | English | DUNDRUM - 3 & 4 CROM; HCR 20 – V3 | X |  |  | X |  |  |  |  |  |  |
| (Baliousis et al., 2015) | 147 | Not stated | Not stated | Forensic psychiatric hospital (RSU) | Forensic psychiatric inpatients | England | English | HoNOS-Secure – Version 2 |  |  |  |  | X |  |  |  |  |  |
| (Bjorkly et al., 2014) | 20 | Not stated | Not stated | Forensic psychiatric hospital - MSU | Forensic psychiatric patients | Norway | Norwegian | HCR 20 – V2 and V3 | X |  |  |  |  |  |  |  |  |  |
| (Braithwaite et al., 2010) | 34 | 37.9 (11.7) | 79 | Risk management  and rehabilitation unit of a civil psychiatric hospital | Psychiatric inpatients admitted to a risk management and rehabilitation unit | Canada | French and English | START - Standard and ‘optimised’ |  | X |  |  |  |  |  |  |  |  |
| (Brewer et al.2016) | 29 | Female (range 23-49); male (21-49) | 52 | Forensic psychiatric hospital - MSU | Forensic psychiatric inpatients | England | English | START - Standard |  | X |  |  |  |  |  |  |  |  |
| **Reference** | **N** | **Age – mean (SD)** | **Sex**  **(% male)** | **Setting** | **Participants** | **Country** | **Language** | **Version(s)** | **HCR-20** | **START** | **CANFOR** | **DUNDRUM** | **HoNOS-S** | **LS/CMI** | **VRS** | **SAPROF** | **SVR-20** | **BEST** |
| (Cabeldue et al., 2018) | 140 | 47.9 (13.2) | 81 | Forensic psychiatric hospital | Forensic psychiatric inpatients found Not Guilty by Reason of Insanity | USA | English | HCR 20 – V3 | X |  |  |  |  |  |  |  |  |  |
| (Cartwright et al., 2018) | 152 | 51.2 (10.0) | 100 | Forensic psychiatric hospital | Civilly committed inpatients | USA | English | START - Standard |  | X |  |  |  |  |  |  |  |  |
| (Castelletti et al., 2015) | 50 | 18-35 (24%); 36-50 (58%); >51 (18%) | 70 | High secure forensic hospital | Forensic psychiatric inpatients | Italy | Italian | CANFOR - Clinical – Staff rated |  |  | X |  |  |  |  |  |  |  |
| (Chakhssi et al., 2010) | 291 | 38.2 (9.5) | 100 | 1 Maximum security hospital | Forensic psychiatric patients (TBS) | The Netherlands | Dutch | BEST - 3 subscales, 70 items |  |  |  |  |  |  |  |  |  | X |
| (Cheng et al., 2019) | 32 | 37.3 | 81 | Forensic psychiatric hospital | Forensic psychiatric inpatients | Canada | English | Version 3 | X |  |  |  |  |  |  |  |  |  |
| (Chu et al., 2011) | 50 | 34.7 (13.9) | 76 | Forensic psychiatric hospital | Forensic psychiatric inpatients | Australia | English | START - Standard |  | X |  |  |  |  |  |  |  |  |
| (Chu et al., 2013) | 66 | 34.4  (13.1) | 80 | Forensic psychiatric hospital - HSU | Forensic, security and civil psychiatric inpatients | Australia | English | START - Risk only |  | X |  |  |  |  |  |  |  |  |
| (Coid et al., 2015) | 387 (out of 409 eligible) | 37.8 (9.7) | 89 | Patients discharged from 32 medium secure units across England and Wales | Community forensic psychiatric patients | England and Wales | English | SAPROF – Standard; HCR 20 – V3 | X |  |  |  |  |  |  | X |  |  |
| (Cook et al., 2016) | 39 | 40 (2.53) | 85 | Forensic psychiatric hospital – MSU, LSU | Forensic psychiatric inpatients | Canada | English | HCR 20 – V3 | X |  |  |  |  |  |  |  |  |  |
| (Coupland & Olver, 2018) | 178 | 32 (9.2) | 100 | Regional psychiatric centre | Treated violent offenders | Canada | English | VRS - Standard |  |  |  |  |  |  | X |  |  |  |
| **Reference** | **N** | **Age – mean (SD)** | **Sex**  **(% male)** | **Setting** | **Participants** | **Country** | **Language** | **Version(s)** | **HCR-20** | **START** | **CANFOR** | **DUNDRUM** | **HoNOS-S** | **LS/CMI** | **VRS** | **SAPROF** | **SVR-20** | **BEST** |
| (Craig et al., 2004) | 139 | Referred to RSU - 36.3 (13.6); non-prison sexual offenders – 41.7 (15.0) | 100 | UK Regional Secure Unit (RSU) outpatient service | 88 sexual offenders referred to RSU, 51 non-prison sexual offenders | United Kingdom | English | SVR-20 - Standard |  |  |  |  |  |  |  |  | X |  |
| (Craig et al., 2006) | 141 | Sexual offenders -37.2 (13.3); Non-sexual violent  offenders - 27.8 (8.2) | Not stated | UK Regional Secure Unit outpatient service | 85 sexual offenders, 46 non-sexual violent offenders | United Kingdom | English | SVR-20 - Standard |  |  |  |  |  |  |  |  | X |  |
| (Davoren et al., 2012)* | 86 | 40.6 (12.8) | 100 | Forensic psychiatric hospital (HSU, MSU, LSU) | Forensic psychiatric inpatients | Ireland | English | DUNDRUM - 3 & 4 CROM; CANFOR - Clinical – Staff rated - Unmet needs |  |  | X | X |  |  |  |  |  |  |
| (Davoren et al., 2013) | 56 | 43.7 (12.8) | Not stated | High, medium and low security hospital | Forensic psychiatric inpatients | Ireland | English | DUNDRUM - 3 & 4 CROM; SAPROF – Standard; START - Standard |  | X |  | X |  |  |  | X |  |  |
| (Davoren et al., 2015) | 97 | 41 (12.3) | 92 | Forensic psychiatric hospital (HSU, MSU, LSU) | Forensic psychiatric inpatients | Ireland | English | DUNDRUM - 3 & 4 CROM & PROM |  |  |  | X |  |  |  |  |  |  |
| (Desmarais et al., 2012) | 120 | 38.0 (11.7) | 100 | Forensic psychiatric hospital | Forensic psychiatric inpatients | Canada | English | START - Standard |  | X |  |  |  |  |  |  |  |  |
| (Desmarais et al., 2010) | 137 | 38.6 (11.4) | 89 | Forensic psychiatric hospital | Forensic psychiatric inpatients appearing before review board | Canada | English | START - Standard |  | X |  |  |  |  |  |  |  |  |
| (de Vogel et al., 2004) | 122 | 24.8 (not stated) | 100 | Dutch forensic psychiatric hospital | Forensic psychiatric inpatients | The Netherlands | Dutch | SVR-20 - Standard |  |  |  |  |  |  |  |  | X |  |
| (de Vries Robbe et al., 2011) | 126 | 31 (7.3) | 100 | Forensic psychiatric hospital | Forensic psychiatric inpatients | The Netherlands | Dutch | SAPROF - Standard |  |  |  |  |  |  |  | X |  |  |
| (de Vries Robbe et al., 2013) | 188 | 32 (7.3) | 100 | Forensic psychiatric hospital | Forensic psychiatric inpatients | The Netherlands | Dutch | SAPROF- Standard |  |  |  |  |  |  |  | X |  |  |
| **Reference** | **N** | **Age – mean (SD)** | **Sex**  **(% male)** | **Setting** | **Participants** | **Country** | **Language** | **Version(s)** | **HCR-20** | **START** | **CANFOR** | **DUNDRUM** | **HoNOS-S** | **LS/CMI** | **VRS** | **SAPROF** | **SVR-20** | **BEST** |
| (de Vries Robbe et al., 2015a) | 83 | 30 (7.5) | 100 | 2 Dutch forensic psychiatric hospitals | Sexual offenders discharged from forensic unit | The Netherlands | Dutch | SVR 20 - Standard; SAPROF – Standard; HCR 20 – Version 3 | X |  |  |  |  |  |  | X | X |  |
| (de Vries Robbe et al., 2015b) | 108 | 33.2 (7.17) | 100 | 2 forensic psychiatric hospitals | Forensic psychiatric inpatients | The Netherlands | Dutch | SAPROF - Standard |  |  |  |  |  |  |  | X |  |  |
| (de Vries Robbe et al., 2016) | 185 | 41(9.7) | 79 | Forensic psychiatric hospital | Forensic psychiatric inpatients | The Netherlands | Dutch | SAPROF - Standard |  |  |  |  |  |  |  | X |  |  |
| (Dickens & O'Shea, 2015) | 217 | 34.1 (15.0) | 75 | Forensic psychiatric hospital – MSU and LSU | Forensic psychiatric inpatients | England | English | START - Standard |  | X |  |  |  |  |  |  |  |  |
| (Dickens and O'Shea, 2017) | 418 | 38.7 ( 14.5) | 67 | Independent forensic psychiatric hospital (MSU/LSU) | Forensic psychiatric inpatients - LSU  (n=321) and MSU (n=97) | England | English | HoNOS-Secure - Version 2 |  |  |  |  | X |  |  |  |  |  |
| (Dickens et al., 2007) | 60 | Median 43 (range  18–70) | 75 | Independent forensic psychiatric hospital MSU/LSU (adult mental health,  adult learning disability, and mental health for older people) | Forensic psychiatric inpatients | England | English | HoNOS-Secure - Version 2 |  |  |  |  | X |  |  |  |  |  |
| (Dickens et al., 2010) | 180 | MH (n=132) 40.4 (10.7); LD (n=48)  32.3 (9.1) | 100 | Independent forensic psychiatric hospital (MSU, LSU and open rehabilitation) | Forensic psychiatric inpatients from mental health (n=132) and learning disability (n=48) pathways | England | English | HoNOS-Secure - Version 2 |  |  |  |  | X |  |  |  |  |  |
| (Dolan & Fullam, 2007) | 136 | 35.5 (9.45) | 100 | Medium secure unit | Forensic psychiatric inpatients | England | English | VRS - Version 2 |  |  |  |  |  |  | X |  |  |  |
| (Dolan et al., 2008) | 147 | 36 (9.42) | 92.5 | Medium secure unit | Forensic psychiatric inpatients | England | English | VRS - Version 2 |  |  |  |  |  |  | X |  |  |  |
| **Reference** | **N** | **Age – mean (SD)** | **Sex**  **(% male)** | **Setting** | **Participants** | **Country** | **Language** | **Version(s)** | **HCR-20** | **START** | **CANFOR** | **DUNDRUM** | **HoNOS-S** | **LS/CMI** | **VRS** | **SAPROF** | **SVR-20** | **BEST** |
| (Douglas & Belfrage, 2014) | 32 | 33.3 (14.2) | 81 | General psychiatric hospital | Forensic and general psychiatric inpatients | Sweden | Swedish | HCR 20 – V3 | X |  |  |  |  |  |  |  |  |  |
| (Doyle et al., 2014) | 387 | Non-violent individuals  38.42 (9.73); violent individuals  34.28 (8.69) | 89 | All forensic patients discharged from  32 MSU | Former forensic psychiatric inpatients | England and Wales | English | HCR 20 – V3 | X |  |  |  |  |  |  |  |  |  |
| (Draycott et al., 2012) | 29 | Not stated | Not stated but assumed to be 100 | High secure hospital DSPD unit | Forensic psychiatric inpatients | England | English | VRS - Standard |  |  |  |  |  |  | X |  |  |  |
| (Eckert et al., 2017) | 139 | Long-term forensic psychiatric care 53.0 (8.1);  Regular forensic psychiatric care 44.0 (11.0) | 100 | 2 Forensic Psychiatric hospitals (HSU) | Forensic psychiatric inpatients | The Netherlands | Dutch | DUNDRUM - 3 & 4 CROM |  |  |  | X |  |  |  |  |  |  |
| (Emmanuel & Campbell, 2009) | 20 | Not stated | 0 | Forensic psychiatric hospital and associated outpatient service | Forensic psychiatric inpatients and outpatients | England | English | CANFOR - Research – staff and user rated |  |  | X |  |  |  |  |  |  |  |
| (Fan, 2015) | 489 | 38.7 (11.0) | 93 | 6 forensic psychiatric hospitals (MSU/LSU) | Forensic psychiatric inpatients | England | English | HoNOS-Secure - Version 2 |  |  |  |  | X |  |  |  |  |  |
| (Finch et al., 2017) | 74 | Not stated | 100 | Forensic psychiatric hospital (MSU/LSU) | Forensic psychiatric inpatients | Australia | English | HoNOS-Secure - Version 2; START - Standard |  | X |  |  | X |  |  |  |  |  |
| (Fox et al., 2015) | 18 | 29.0 (9.0) | 0 | Forensic psychiatric hospital -Dialectical Behavioural Therapy unit | Forensic psychiatric inpatients | England | English | HoNOS-Secure - Version 2 |  |  |  |  | X |  |  |  |  |  |
| (Girardi et al., 2019) | 28 | Non violent individuals 30.5 (10.6); violent individuals  33.2 (11.4) | 100 | Forensic psychiatric hospital | Forensic psychiatric inpatients | England | English | HCR 20 – V3 | X |  |  |  |  |  |  |  |  |  |
| **Reference** | **N** | **Age – mean (SD)** | **Sex**  **(% male)** | **Setting** | **Participants** | **Country** | **Language** | **Version(s)** | **HCR-20** | **START** | **CANFOR** | **DUNDRUM** | **HoNOS-S** | **LS/CMI** | **VRS** | **SAPROF** | **SVR-20** | **BEST** |
| (Gray et al., 2011) | 44 | 40.2 (14.7) | 64 | 1 forensic and 2 general wards in a psychiatric hospital | Forensic and civil psychiatric inpatients | Wales | English | START - Standard with 5 point SPJ ratings |  | X |  |  |  |  |  |  |  |  |
| (Green et al., 2016) | 124 | 45.2 (13.0) | 81 | Forensic psychiatric hospital | Forensic psychiatric patients | USA | English | HCR 20 – V3 | X |  |  |  |  |  |  |  |  |  |
| (Grevatt et al., 2004) | 44 | 44 (range 19-65) | 100 | Independent secure unit | Forensic psychiatric inpatients | England | English | VRS - Standard |  |  |  |  |  |  | X |  |  |  |
| (Griffiths et al., 2018) | 347 | Not secluded 35.2 (12.8); Secluded 30.8 (10.7) | Not secluded 67; Secluded 59 | 4 forensic psychiatric hospitals (MSU/LSU) | Forensic psychiatric inpatients | England | English | HoNOS-Secure - Version 2 |  |  |  |  | X |  |  |  |  |  |
| (Grossi et al., 2019) | 169 | 44.2 (13.2) | 83 | Forensic psychiatric hospital | Forensic psychiatric inpatients | USA | English | HCR 20 – V3 | X |  |  |  |  |  |  |  |  |  |
| (Haines et al., 2018) | 261 | 39.4 (13.1) | 62 | 1 Medium Secure Unit, 4 Low Secure Units, 9 general acute wards, 6 Community Mental Health Teams | Forensic psychiatric inpatients (n=55); general inpatient (n=100); general community patients (n=106) | England | English | SAPROF - Standard |  |  |  |  |  |  |  | X |  |  |
| (Hogan & Olver, 2016) | 99 | 36.7 | 86 | Maximum security unit at a psychiatric hospital | Forensic psychiatric inpatients | Canada | English | VRS – Standard; START – Standard; HCR 20 – V3 | X | X |  |  |  |  | X |  |  |  |
| (Horgan et al., 2019) | 33 | 38.1 (11.4) | 100 | Medium secure unit | Forensic psychiatric inpatients | England | English | VRS - Standard |  |  |  |  |  |  | X |  |  |  |
| (Howden et al., 2018) | 25 | 32 (8.1) | 100 | Medium secure unit | Forensic psychiatric inpatients | England | English | VRS - Standard |  |  |  |  |  |  | X |  |  |  |
| (Inett et al., 2014) | 27 | 39 | 100 | Forensic psychiatric hospital – Intellectual Disability LSU | Forensic psychiatric inpatients | England | English | START – Standard, plus 2 case specific items |  | X |  |  |  |  |  |  |  |  |
| **Reference** | **N** | **Age – mean (SD)** | **Sex**  **(% male)** | **Setting** | **Participants** | **Country** | **Language** | **Version(s)** | **HCR-20** | **START** | **CANFOR** | **DUNDRUM** | **HoNOS-S** | **LS/CMI** | **VRS** | **SAPROF** | **SVR-20** | **BEST** |
| (Jung et al., 2012) | 219 (LS/CMI –n=138) | 29.2 (9.5) -  for whole sample (n=219) | 81 - for whole sample (n=219) | Forensic  psychiatric outpatient clinic | Offenders referred for a pre-sentence psychiatric evaluation following a criminal conviction | Canada | English | LS/CMI - Standard version |  |  |  |  |  | X |  |  |  |  |
| (Jung et al., 2013) | 102 | 30.8 (11.7) | 89 | Forensic psychiatric hospital | Offenders referred for a pre-sentence psychiatric evaluation following a criminal conviction | Canada | English | LS/CMI - Standard version |  |  |  |  |  | X |  |  |  |  |
| (Kashiwagi et al., 2018) | 95 | 45.73 (14.12) | 87 | Forensic psychiatric unit | Forensic psychiatric inpatients | Japan | Japanese | SAPROF - Standard |  |  |  |  |  |  |  | X |  |  |
| (Lam, 2015) | 110 | 37.3 (11.5) | 100 | Forensic psychiatric hospital – Maximum security | Forensic psychiatric inpatients | USA | English | START - Standard |  | X |  |  |  |  |  |  |  |  |
| (Langton, 2011; Langton et al., 2009) | 44 | 34.41 (8.47) | 100 | High secure hospital DSPD unit | Forensic psychiatric inpatients | England | English | VRS - Standard |  |  |  |  |  |  | X |  |  |  |
| (Lewis et al., 2013)@ | 150 | 30.2 (range 18.1-52.0) | 100 | Regional psychiatric centre | Forensic psychiatric patients | Canada | English | VRS - Standard |  |  |  |  |  |  | X |  |  |  |
| (Long et al., 2010) | 60 | 31.3 (8.9) | 0 | Independent forensic psychiatric hospital MSU | Forensic psychiatric inpatients | England | English | HONOS-Secure - Version 2 |  |  |  |  | X |  |  |  |  |  |
| (Long & Dolley, 2012) | 70 | 30.4 (7.6) | 0 | Independent forensic psychiatric hospital - MSU | Forensic psychiatric inpatients | England | English | CANFOR - Clinical version – staff rated -Psychotic symptoms; psychological distress |  |  | X |  |  |  |  |  |  |  |
| (Long et al., 2011a) | 24 | 32.6 (9.2) | 0 | Independent forensic psychiatric hospital - MSU | Forensic psychiatric inpatients | England | English | HONOS-Secure - Version 2 Item 9 – ‘Problems with relationships’ only |  |  |  |  | X |  |  |  |  |  |
| (Long et al., 2011b) | 70 | 31.3 (8.5) | 0 | Independent forensic psychiatric hospital MSU | Forensic psychiatric inpatients | England | English | HONOS-Secure - Version 2 -Security scale only |  |  |  |  | X |  |  |  |  |  |
| **Reference** | **N** | **Age – mean (SD)** | **Sex**  **(% male)** | **Setting** | **Participants** | **Country** | **Language** | **Version(s)** | **HCR-20** | **START** | **CANFOR** | **DUNDRUM** | **HoNOS-S** | **LS/CMI** | **VRS** | **SAPROF** | **SVR-20** | **BEST** |
| (Longdon et al., 2017) | 108 | 34.3 (10.5) | 100 | Forensic psychiatric hospital | Forensic psychiatric inpatients | England | English | HoNOS-Secure - Version 2 -Item 11 -‘Problems with living  conditions’ omitted. |  |  |  |  | X |  |  |  |  |  |
| (Marriott et al., 2017) | 527 | 40.4 (15.8) | 74 | Forensic psychiatric hospital | Forensic psychiatric inpatients | England | English | START - Standard |  | X |  |  |  |  |  |  |  |  |
| (Mastromanno et al., 2018) | 40 | 30.3 (9.2) | 32 | Forensic psychiatric hospital | Forensic psychiatric patients | Australia | English | HCR 20 – V3 | X |  |  |  |  |  |  |  |  |  |
| (Murphy, 2007) | 30 | 37.4 (8.4) | 100 | Forensic psychiatric hospital - HSU | Forensic psychiatric inpatients | England | English | HONOS-Secure - Version 2? |  |  |  |  | X |  |  |  |  |  |
| (Nicholls et al., 2006) | 137 | Men 38.3 (11.3); women  39.6 (11.4) | 89 | Forensic psychiatric hospital | Forensic psychiatric inpatients | Canada | English | START - Version with 6-point rating scale. |  | X |  |  |  |  |  |  |  |  |
| (Nicholls et al., 2011) | 1057# | Men – 39 (12.6); women - 40 (11.9) | 88 | Forensic psychiatric service | Forensic psychiatric inpatients and outpatients | Canada | English | START - Standard |  | X |  |  |  |  |  |  |  |  |
| (Nonstad et al., 2010) | 47 | 36 (range 20–60) | 83 | Forensic psychiatric hospital - HSU | Forensic psychiatric inpatients | Norway | Norwegian | START - Standard |  | X |  |  |  |  |  |  |  |  |
| (O'Dwyer et al., 2011)* | 95 | 40.9 (95% CI  38.4-43.5) | 92 | Forensic psychiatric hospital (HSU, MSU, LSU) | Forensic psychiatric inpatients | Ireland | English | DUNDRUM - 3 & 4 CROM; CANFOR Clinician –Staff rated |  |  | X | X |  |  |  |  |  |  |
| (O'Shea & Dickens, 2015) | 827 | 38.5 (16.7) | 72 | Forensic psychiatric hospital | Forensic psychiatric inpatients | England | English | START - Standard |  | X |  |  |  |  |  |  |  |  |
| (O'Shea & Dickens, 2016) | 84 | 34.2 (14.2) | 73 | Forensic psychiatric hospital | Forensic psychiatric inpatients | England | English | START - Standard |  | X |  |  |  |  |  |  |  |  |
| (O'Shea et al., 2016) | 200 | 34.3 (15.2) | 75 | Forensic psychiatric hospital | Forensic psychiatric inpatients | England | English | START - Standard |  | X |  |  |  |  |  |  |  |  |
| (Olver et al., 2013)@ | 152 | 30.5 (range 18.1-55.9) | 100 | Regional psychiatric centre | Forensic psychiatric patients | Canada | English | VRS - Standard |  |  |  |  |  |  | X |  |  |  |
| **Reference** | **N** | **Age – mean (SD)** | **Sex**  **(% male)** | **Setting** | **Participants** | **Country** | **Language** | **Version(s)** | **HCR-20** | **START** | **CANFOR** | **DUNDRUM** | **HoNOS-S** | **LS/CMI** | **VRS** | **SAPROF** | **SVR-20** | **BEST** |
| (Penney et al., 2016) | 87 | 36.44 (9.82) | 84 | Forensic psychiatric hospital | Forensic psychiatric patients | Canada | English | HCR 20 – V3 | X |  |  |  |  |  |  |  |  |  |
| (Persson et al., 2017) | 200 | 31.0 (IQR= 25.0-41.5) | 87 | Forensic psychiatric hospital | Forensic psychiatric inpatients | Sweden | Swedish | SAPROF –Standard; HCR 20 – V3 | X |  |  |  |  |  |  | X |  |  |
| (Pillay et al., 2008) | 70 | 42.6 (13.3) | 100 | Forensic psychiatric hospital (HSU, MSU, LSU) | Forensic psychiatric inpatients | Ireland | English | HoNOS-Secure - Version 2; CANFOR Research – Staff and user rated |  |  | X |  | X |  |  |  |  |  |
| (Puzzo et al., 2019) | 48 | 38.8 (9.6) | 100 | Forensic psychiatric hospital - HSU | Forensic psychiatric inpatients | England | English | HCR 20 –V3 | X |  |  |  |  |  |  |  |  |  |
| (Quinn et al., 2013) | 80 | 38.4 (10.1) | 74 | Forensic psychiatric hospital - MSU | Forensic psychiatric inpatients | England | English | HoNOS-Secure - Version 2; START - Standard, plus 2 case specific items |  | X |  |  | X |  |  |  |  |  |
| (Ribeiro et al., 2015) | 45 | 34.7 (range 19 to 73) | 0 | Forensic psychiatric hospital - MSU | Forensic psychiatric inpatients | England | English | HoNOS-Secure - Version 2 |  |  |  |  | X |  |  |  |  |  |
| (Richter et al., 2018) | 69 | 39.7 (11.1) | Not stated | Forensic psychiatric hospital (HSU, MSU, LSU) | Forensic psychiatric inpatients | Ireland | English | DUNDRUM - 3 CROM |  |  |  | X |  |  |  |  |  |  |
| (Romeva et al., 2010) | 90 | Not stated | 97 | Psychiatric ward of 4 prisons | Mentally ill prisoners | Spain | Spanish | CANFOR - Clinical - Spanish |  |  | X |  |  |  |  |  |  |  |
| (Ross et al., 2007; Ross et al., 2008; Ross et al., 2012) | 231 | Men = 38.0 (10.3)  Women = 34.7 (9.2) | 85.7 | Various secure hospitals in four countries | Forensic psychiatric patients | United Kingdom (58), Germany (89), The Netherlands (53) and Norway (31) | English, German, Dutch and Norwegian | 6 subscales, 150 items |  |  |  |  |  |  |  |  |  | X |
| (Segal et al., 2010) | 50 | 35.7 (9.9) | Not stated | Forensic psychiatric hospital | Forensic psychiatric inpatients | Australia | English | HoNOS-Secure Version 2 |  |  |  |  | X |  |  |  |  |  |
| (Shinkfield and Ogloff, 2016) | 118 | Not stated | 85 | Forensic psychiatric hospital | Forensic psychiatric inpatients | Australia | English | HoNOS-Secure Version 2 |  |  |  |  | X |  |  |  |  |  |
| **Reference** | **N** | **Age – mean (SD)** | **Sex**  **(% male)** | **Setting** | **Participants** | **Country** | **Language** | **Version(s)** | **HCR-20** | **START** | **CANFOR** | **DUNDRUM** | **HoNOS-S** | **LS/CMI** | **VRS** | **SAPROF** | **SVR-20** | **BEST** |
| (Sugarman et al., 2009) | Not stated | Not stated | Not stated | Independent psychiatric hospital (adult mental health,  learning disability, adolescent mental illness, and mental health for older people) | Forensic, adolescent, learning disability, acquired brain injury psychiatric inpatients | England | English | HoNOS-Secure Version 2 |  |  |  |  | X |  |  |  |  |  |
| (Talina et al., 2013) | 143 | Males 39.7 (12.8);  Females 35.2 (10.0) | 76 | Forensic ward in a general psychiatric hospital, psychiatric ward in a prison-hospital, 2 prison psychiatric outpatient clinics | Forensic psychiatric inpatients and prisoners | Portugal | Portuguese | CANFOR - Research version |  |  | X |  |  |  |  |  |  |  |
| (Thomas et al., 2008) | 77 | 38.75 (11.16) | 69 | High security forensic psychiatric hospital and medium security forensic psychiatric hospital | Forensic psychiatric inpatients | England | English | CANFOR - Clinical version – staff and service users |  |  | X |  |  |  |  |  |  |  |
| (Trizna & Adamowski, 2016) | 93 | 44.7 (13.7) | 100 | Forensic psychiatric hospital – MSU, LSU | Forensic psychiatric inpatients | Poland | Polish | CANFOR - Research |  |  | X |  |  |  |  |  |  |  |
| (Troquete et al., 2015) | 310 | 40 (11) | 94 | 3 outpatient forensic services | Forensic psychiatric outpatients | The Netherlands | Dutch | START - Standard |  | X |  |  |  |  |  |  |  |  |
| (Tully et al., 2019) | 50 | 37.5 (11.2) | 0 | Forensic psychiatric hospital - MSU | Forensic psychiatric inpatients | England | English | HoNOS-Secure - Version 2 |  |  |  |  | X |  |  |  |  |  |
| (Viljoen et al., 2011) | 48 | 37 (11.0) | 0 | Forensic psychiatric service | Forensic psychiatric outpatients | Canada | English | START - Standard |  | X |  |  |  |  |  |  |  |  |
| (Walker et al., 2019) | 245  (Detailed data for 75) | 40 (10.67) | 98 | 1 HSU and 1 MSU/LSU | Forensic psychiatric  Patients | Scotland and Ireland | English | BEST- 6 subscales, 150 items |  |  |  |  |  |  |  |  |  | X |
| (Whittington et al., 2014) | 50 | 38.6 (range 19–65) | 88 | Forensic psychiatric hospital - MSU | Forensic psychiatric inpatients | England | English | START - Standard |  | X |  |  |  |  |  |  |  |  |
| (Wilson et al., 2010)$ | 30 | 37.1 (12.1) | 100 | Forensic psychiatric hospital - MSU | Forensic psychiatric inpatients | Canada | English | START - Standard |  | X |  |  |  |  |  |  |  |  |
| (Wilson et al., 2013)$ | 30 | 37.1 (12.1) | 100 | Forensic psychiatric hospital - MSU | Forensic psychiatric inpatients | Canada | English | START - Standard |  | X |  |  |  |  |  |  |  |  |
| **Reference** | **N** | **Age – mean (SD)** | **Sex**  **(% male)** | **Setting** | **Participants** | **Country** | **Language** | **Version(s)** | **HCR-20** | **START** | **CANFOR** | **DUNDRUM** | **HoNOS-S** | **LS/CMI** | **VRS** | **SAPROF** | **SVR-20** | **BEST** |
| (Wilson et al., 2014) | 47 | 32.9 (9.84) | 100 | Medium secure unit | Forensic psychiatric inpatients with personality disorders | England | English | VRS - Standard |  |  |  |  |  |  | X |  |  |  |
| (Wong & Gordon, 2006) | 918 | 38.8 (9.59) | 100 | Federal prison, local prison and forensic psychiatric unit | 558 federal prisoners, 30 provincial prisoners, 330 forensic psychiatric inpatients | Canada | English | VRS - Standard |  |  |  |  |  |  | X |  |  |  |
| (Woods, 1999; Woods & Reed, 1999; Woods et al, 1999; Woods et al. 2001a; Woods et al. 2001b; Woods et al. 2001c; Woods et al. 2003a; Woods et al. 2003b; Woods et al. 2004; Woods et al. 2005) | 503 | Not reported | 87.3 | 2 HSUs | Forensic psychiatric patients | England | English | BEST - 3 subscales, 70 items |  |  |  |  |  |  |  |  |  | X |
| (Yoon et al., 2011) | 30 | 48 (12.0) | 100 | Forensic outpatient programme | Sexual offenders on probation or with parole supervision | Germany | German | SAPROF – Standard, SVR-20 - Standard |  |  |  |  |  |  |  | X | X |  |

*Note:* This table displays all 115 sources included in the systematic review. Where more than one reference is provided, the relevant study was published in more than one article.

# - unclear if this number refers to assessments or participants.

*/@ - there appears to be some overlap in the study populations

Abbreviations:

HCR-20 – Historical, Clinical, Risk 20

START - Short-Term Assessment of Risk and Treatability

CANFOR - Camberwell Assessment of Need – Forensic

DUNDRUM - Dangerousness, Understanding, Recovery and Urgency Manual

HoNOS-Secure - Health of the Nation Outcome Scale Secure

LS/CMI - Level of Service: Case Management Inventory

VRS - Violence Risk Scale

SAPROF - Structured Assessment of Protective Factors for Violence Risk

SVR-20 - Sexual Violence Risk 20

BEST - Behavioural Status Index

HSU – High Secure Unit

MSU – Medium Secure Unit

LSU – Low Secure Unit

RSU – Regional Secure Unit (usually synonymous with a MSU)

IQR – Interquartile range

References:

Abidin, Z., Davoren, M., Naughton, L., Gibbons, O., Nulty, A. & Kennedy, H. 2013. Susceptibility (risk and protective) factors for in-patient violence and self-harm: Prospective study of structured professional judgement instruments START and SAPROF, DUNDRUM-3 and DUNDRUM-4 in forensic mental health services. BMC Psychiatry, 13, 197.

Abou-Sinna, R. & Luebbers, S. 2012. Validity of assessing people experiencing mental illness who have offended using the Camberwell Asessment of Need-Forensic and Health of the Nation Outcome Scales-Secure. International Journal of Mental Health Nursing, 21, 462-70.

Adams, J., Thomas, S., Mackinnon, T. & Eggleton, D. 2018. The risks, needs and stages of recovery of a complete forensic patient cohort in an Australian state. BMC Psychiatry, 18, 35.

Baliousis, M., Huband, N., Duggan, C., McCarthy, L. & Vollm, B. 2015. Development and validation of a treatment progress scale for personality disordered offenders. Personality and Mental Health, 9, 107-123.

Bjorkly, S., Eidhammer, G. & Selmer L. 2014. Concurrent validity and clinical utility of the HCR-20 V3 compared with the HCR-20 in forensic mental health nursing: similar tools but improved method. Journal of Forensic Nursing, 10, 234-42.

Braithwaite, E., Charette, Y., Crocker, A. & Reyes, A. 2010. The predictive validity of clinical ratings of the Short-Term Assessment of Risk and Treatability (START). International Journal of Forensic Mental Health, 9, 271-281.

Brewer, R., Pomroy, L., Wells, M. & Ratcliffe J. 2016. The Short Dynamic Risk Scale (SDRS) vs START: does either have a relationship with recordings of risk? Journal of Intellectual Disabilities and Offending Behaviour, 7, 202-212.

Cabeldue, M., Green, D., Griswold, H., Schneider, M., Smith, J., Belfi, B. & Kunz, M. 2018. Using the HCR-20 (V3) to differentiate insanity acquittees based on opinions of readiness for transfer. Journal of the American Academy of Psychiatry and the Law, 46, 339-350.

Cartwright, J., Desmarais, S., Hazel, J., Griffith, T. & Azizian, A. 2018. Predictive validity of HCR-20, START, and Static-99R assessments in predicting institutional aggression among sexual offenders. Law and Human Behavior, 42, 13-25.

Castelletti, L., Lasalvia, A., Molinari, E., Thomas, S., Stratico, E & Bonetto, C. 2015. A standardised tool for assessing needs in forensic psychiatric population: Clinical validation of the Italian CANFOR, staff version. Epidemiology and Psychiatric Sciences, 24, 274-281.

Chakhssi, F., de Ruiter, C. & Bernstein, D. 2010. Reliability and validity of the Dutch version of the Behavioural Status Index: A nurse-rated forensic assessment tool. Assessment, 17, 58-69.

Cheng, J., Haag, A. & Olver, M. 2019. Predictors of Historical Clinical Risk Management-20 Version 3 (HCR-20:V3) summary risk ratings. Psychiatry, Psychology and Law, 26, 682-692.

Chu, C., Thomas, S., Ogloff, J. & Daffern, M. 2011. The predictive validity of the Short-Term Assessment of Risk and Treatability (START) in a secure forensic hospital: Risk factors and strengths. International Journal of Forensic Mental Health, 10, 337-345.

Chu, C., Thomas, S., Ogloff, J. & Daffern, M. 2013. The short- to medium-term predictive accuracy of static and dynamic risk assessment measures in a secure forensic hospital. Assessment, 20, 230-241.

Coid, J., Kallis, C., Doyle, M., Shaw, J. & Ullrich, S. 2015. Identifying causal risk factors for violence among discharged patients. PLoS ONE, 10, e0142493.

Cook, A., Moulden, H., Mamak, M., Lalani, S., Messina, K. & Chaimowitz, G. 2016. Validating the Hamilton Anatomy of Risk Management-Forensic Version and the Aggressive Incidents Scale. Assessment, 25, 432-445.

Coupland, R. & Olver, M. 2018. Assessing dynamic violence risk in a high-risk treated sample of violent offenders. Assessment, 27, 1886-1900.

Craig, L., Browne, K. & Stringer, I. 2004. Comparing sex offender risk assessment measures on a UK sample. International Journal of Offender Therapy & Comparative Criminology, 48, 7-27.

Craig, L., Beech, A. & Browne, K. 2006. Cross-validation of the Risk Matrix 2000 sexual and violent scales. Journal of Interpersonal Violence. 2006, 21, 612-633.

Davoren, M., O'Dwyer, S., Abidin, Z., Naughton, L., Gibbons, O., Doyle, E., McDonnell, K. Monks, S. & Kennedy, H. 2012.. Prospective in-patient cohort study of moves between levels of therapeutic security: The DUNDRUM-1 triage security, DUNDRUM-3 programme completion and DUNDRUM-4 recovery scales and the HCR-20. BMC Psychiatry,12, 80.

Davoren, M., Abidin, Z., Naughton, L., Gibbons, O., Nulty, A., Wright, B. & Kennedy, H. 2013. Prospective study of factors influencing conditional discharge from a forensic hospital: The DUNDRUM-3 programme completion and DUNDRUM-4 recovery structured professional judgement instruments and risk. BMC Psychiatry, 13, 185.

Davoren, M., Hennessy, S., Conway, C., Marrinan, S., Gill, P. & Kennedy, H. 2015. Recovery and concordance in a secure forensic psychiatry hospital - the self rated DUNDRUM-3 programme completion and DUNDRUM-4 recovery scales. BMC Psychiatry, 15, 61.

Desmarais, S., Nicholls, T., Read, D. & Brink, J. 2010. Confidence and accuracy in assessments of short-term risks presented by forensic psychiatric patients. Journal of Forensic Psychiatry and Psychology, 21, 1-22.

Desmarais, S., Nicholls, T., Wilson, C. & Brink, J. 2012. Using dynamic risk and protective factors to predict inpatient aggression: Reliability and validity of START assessments. Psychological Assessment, 24, 685-700.

de Vogel, V., de Ruiter, C., van Beek, D. & Mead, G. 2004. Predictive validity of the SVR-20 and Static-99 in a Dutch sample of treated sex offenders. Law and Human Behavior, 28, 235-251.

de Vries Robbe, M., de Vogel, V. & de Spa, E. 2011. Protective factors for violence risk in forensic psychiatric patients: A retrospective validation study of the SAPROF. International Journal of Forensic Mental Health, 10, 178-186.

de Vries Robbe, M., de Vogel, V. & Douglas, K. 2013. Risk factors and protective factors: A two-sided dynamic approach to violence risk assessment. Journal of Forensic Psychiatry and Psychology, 24, 440-457.

de Vries Robbe, M., de Vogel, V., Koster, K. & Bogaerts, S. 2015a. Assessing protective factors for sexually violent offending with the SAPROF. Sexual Abuse, 27, 51-70.

de Vries Robbe, M., de Vogel, V., Douglas, K. & Nijman, H. 2015b. Changes in dynamic risk and protective factors for violence during inpatient forensic psychiatric treatment: Predicting reductions in postdischarge community recidivism. Law and Human Behavior, 39, 53-61.

de Vries Robbe, M., de Vogel, V., Wever, E., Douglas, K. & Nijman, H. 2016. Risk and protective factors for inpatient aggression. Criminal Justice and Behavior, 43, 1364-1385.

Dickens, G. & O'Shea, L. 2015. How short should short-term risk assessment be? Determining the optimum interval for START reassessment in a secure mental health service. Journal of Psychiatric and Mental Health Nursing, 22, 397-406.

Dickens, G. & O'Shea, L. 2017. Reliable and clinically significant change in outcomes for forensic mental health inpatients: Use of the HoNOS-Secure. International Journal of Forensic Mental Health, 16, 161-171.

Dickens, G., Sugarman, P. & Walker, L. 2007. HoNOS-secure: A reliable outcome measure for users of secure and forensic mental health services. Journal of Forensic Psychiatry and Psychology, 18, 507-514.

Dickens, G., Sugarman, P., Picchioni, M. & Long, C. 2010. HoNOS-Secure: Tracking risk and recovery for men in secure care. The British Journal of Forensic Practice, 12, 36-46.

Dolan, M. & Fullam, R. 2007. The validity of the Violence Risk Scale second edition (VRS-2) in a British forensic inpatient sample. Journal of Forensic Psychiatry and Psychology, 18, 381-393.

Dolan, M., Fullam, R., Logan, C. & Davies, G. 2008. The Violence Risk Scale Second Edition (VRS-2) as a predictor of institutional violence in a British forensic inpatient sample. Psychiatry Research, 158, 55-65.

Douglas, K. & Belfrage, H. 2014. Interrater reliability and concurrent validity of the HCR-20 Version 3. International Journal of Forensic Mental Health, 13, 130-139.

Doyle, M., Power, L., Coid, J., Kallis, C., Ullrich, S. & Shaw, J. 2014. Predicting post-discharge community violence in England and Wales using the HCR-20 (V3). International Journal of Forensic Mental Health, 13, 140-147.

Draycott, S., Kirkpatrick, T. & Askari, R. 2012. An idiographic examination of patient progress in the treatment of dangerous and severe personality disorder: A reliable change index approach. Journal of Forensic Psychiatry and Psychology, 23, 108-124.

Eckert, M., Schel, S., Kennedy, H. & Bulten, B. 2017. Patient characteristics related to length of stay in Dutch forensic psychiatric care. Journal of Forensic Psychiatry and Psychology, 28, 863-880.

Emmanuel, M. & Campbell, M. 2009. Comparing staff and client perceptions of needs in a British single-sex medium secure unit. Psychological Reports, 104, 1033-1034.

Fan, C. 2015. Psychometric properties and descriptive characteristics of clients by using two theory-based assessments. PhD. thesis, University of Illinois, Chicago.

Finch, B., Gilligan, D., Halpin, S. & Valentine, M. 2017. The short- to medium-term predictive validity of static and dynamic risk-of-violence measures in medium- to low-secure forensic and civil inpatients. Psychiatry, Psychology and Law, 24, 410-427.

Fox, E., Krawczyk, K., Staniford, J. & Dickens, G. 2015. A service evaluation of a 1-Year dialectical behaviour therapy programme for women with borderline personality disorder in a low secure unit. Behavioural and Cognitive Psychotherapy, 43, 676-691.

Girardi, A., Hancock-Johnson, E., Thomas, C. & Wallang, P. 2019. Assessing the risk of inpatient violence in autism spectrum disorder. Journal of the American Academy of Psychiatry and the Law, 47, 427-436.

Gray, N., Benson, R., Craig, R., Davies, H., Fitzgerald, S., Huckle, P., Maggs, R., Taylor, J., Trueman, M., Williams, T. & Snowden, R. 2011. The Short-Term Assessment of Risk and Treatability (START): A prospective study of inpatient behavior. International Journal of Forensic Mental Health, 10, 305-313.

Green, D., Schneider, M., Griswold, H., Belfi, B., Herrera, M. & DeBlasi, A. 2016. A comparison of the HCR-20(V3) among male and female insanity acquittees: A retrospective file study. International Journal of Forensic Mental Health, 15, 48-64.

Grevatt, M., Thomas-Peter, B. & Hughes, G. 2004. Violence, mental disorder and risk assessment: Can structured clinical assessments predict the short-term risk of inpatient violence? Journal of Forensic Psychiatry and Psychology, 15, 278-292.

Griffiths, C., Roychowdhury, A. & Girardi, A. 2018. Seclusion: the association with diagnosis, gender, length of stay and HoNOS-secure in low and medium secure inpatient mental health service. Journal of Forensic Psychiatry and Psychology, 29, 1-18.

Grossi, L., Green, D., Griswold, H., Cabeldue, M. & Belfi, B. 2019. Assessing inpatient victimization risk among insanity acquittees using the HCR-20(V3). Journal of the American Academy of Psychiatry and the Law, 47, 286-298.

Haines, A., Brown, A., Javaid, S., Khan, F., Noblett, S., Omodunbi, O., Sadiq, K., Zaman, W. & Whittington, R. 2018. Assessing protective factors for violence risk in U.K. general mental health services using the Structured Assessment of PROtective Factors. International Journal of Offender Therapy and Comparative Criminology, 62, 3965-3983.

Hogan, N. & Olver, M. 2016. Assessing risk for aggression in forensic psychiatric inpatients: An examination of five measures. Law and Human Behavior, 40, 233-243.

Horgan, H., Charteris, C. & Ambrose, D. 2019. The Violence Reduction Programme: An exploration of post-treatment risk reduction in a specialist medium-secure unit. Criminal Behaviour and Mental Health, 29, 286-295.

Howden, S., Midgley, J. & Hargate, R. 2018. Violent offender treatment in a medium secure unit. Journal of Forensic Practice, 20, 102-111.

Inett, A., Wright, G., Roberts, L. & Sheeran, A. 2014. Predictive validity of the START with intellectually disabled offenders. Journal of Forensic Practice, 16, 78-88.

Jung, S., Daniels, M., Friesen, M. & Ledi, D. 2012. An examination of convergent constructs among Level of Service measures and other measures. Journal of Forensic Psychiatry and Psychology, 23, 601-619.

Jung, S., Ledi, D. & Daniels, M. 2012. Evaluating the concurrent validity of the HCR-20 scales. Journal of Risk Research, 16, 697-711.

Kashiwagi, H., Kikuchi, A., Koyama, M., Saito, D. & Hirabayashi, N. 2018. Strength-based assessment for future violence risk: a retrospective validation study of the Structured Assessment of PROtective Factors for violence risk (SAPROF) Japanese version in forensic psychiatric inpatients. Annals of General Psychiatry, 17, 5.

Lam, J. 2015. Use of the Short Term Assessment of Risk and Treatability in a forensic facility: Examining the impact of suicide behavior on multiple risk outcomes. PhD. thesis, Fordham University, New York City.

Langton, C. 2011. Personality traits and dynamic variables associated with types of aggression in high security forensic psychiatric inpatients. PhD. thesis, University of Toronto, Toronto.

Langton, C., Hogue, T., Daffern, M., Mannion, A. & Howells, K. 2009. Prediction of institutional aggression among personality disordered forensic patients using actuarial and structured clinical risk assessment tools: prospective evaluation of the HCR-20, VRS, Static-99, and Risk Matrix 2000. Psychology, Crime and Law, 15, 635-659.

Lewis, K., Olver, M. & Wong, S. 2013. The Violence Risk Scale: predictive validity and linking changes in risk with violent recidivism in a sample of high-risk offenders with psychopathic traits. Assessment, 20, 150-164.

Long, C., Dickens, G., Sugarman, P., Craig, L., Mochty, U. & Hollin, C. 2010. Tracking risk profiles and outcome in a medium secure service for women: Use of the HoNOS-Secure. International Journal of Forensic Mental Health, 9, 215-225.

Long, C. & Dolley, O. 2012. Factors predictive of length of stay for women in medium secure settings. Journal of Psychiatric and Mental Health Nursing, 19, 870-874.

Long, C., Fulton, B., Dolley, O. & Hollin, C. 2011a. Social problem-solving interventions in medium secure settings for women. Medicine, Science and the Law, 51, 215-219.

Long, C., Dolley, O. & Hollin, C. 2011b. Women in medium secure care: Tracking treatment progress for changes in risk profiles and treatment engagement. Journal of Psychiatric and Mental Health Nursing, 18, 425-431.

Longdon, L., Edworthy, R., Resnick, J., Byrne, A., Clarke, M., Cheung, N. & Khalifa, N. 2017. Patient characteristics and outcome measurement in a low secure forensic hospital. Criminal Behaviour and Mental Health, 28, 255-269.

Marriott, R., O'Shea, L., Picchioni, M. & Dickens, G. 2017. Predictive validity of the Short-Term Assessment of Risk and Treatability (START) for multiple adverse outcomes: The effect of diagnosis. Psychiatry Research, 256, 435-443.

Mastromanno, B., Brookstein, D., Ogloff, J., Campbell, R., Chu, C. & Daffern, M. 2018. Assessing change in dynamic risk factors in forensic psychiatric inpatients: Relationship with psychopathy and recidivism. Journal of Forensic Psychiatry and Psychology, 29, 323-336.

Murphy, D. 2007. Theory of mind functioning in mentally disordered offenders detained in high security psychiatric care: Its relationship to clinical outcome, need and risk. Criminal Behaviour and Mental Health, 17, 300-311.

Nicholls, T., Brink, J., Desmarais, S., Webster, C. & Martin, M. 2006. The Short-Term Assessment of Risk and Treatability (START): A prospective validation study in a forensic psychiatric dample. Assessment, 13, 313-327.

Nicholls, T., Petersen, K., Brink, J. & Webster, C. 2011. A clinical and risk profile of forensic psychiatric patients: Treatment team STARTs in a Canadian service. International Journal of Forensic Mental Health, 10, 187-199.

Nonstad, K., Nesset, M., Kroppan, E., Pedersen, T., Nottestad, J., Almvik, R. & Palmstierna, T. 2010. Predictive validity and other psychometric properties of the Short-Term Assessment of Risk and Treatability (START) in a Norwegian high secure hospital. International Journal of Forensic Mental Health, 9, 294-299.

O'Dwyer, S., Davoren, M., Abidin, Z., Doyle, E., McDonnell, K. & Kennedy, H. 2011.The DUNDRUM Quartet: Validation of structured professional judgement instruments DUNDRUM-3 assessment of programme completion and DUNDRUM-4 assessment of recovery in forensic mental health services. BMC Research Notes, 4, 229.

O'Shea, L. & Dickens, G. 2015. Predictive validity of the START for unauthorised leave and substance abuse in a secure mental health setting: a pseudo-prospective cohort study. International Journal of Nursing Studies, 52, 970-979.

O'Shea, L. & Dickens, G. 2016. Role of assessment components and recent adverse outcomes in risk estimation and prediction: Use of the Short Term Assessment of Risk and Treatability (START) in an adult secure inpatient mental health service. Psychiatry Research, 240, 398-405.

O'Shea, L., Picchioni, M. & Dickens, G. 2016. The predictive validity of the Short-Term Assessment of Risk and Treatability (START) for multiple adverse outcomes in a secure psychiatric inpatient setting. Assessment, 23, 150-162.

Olver, M., Lewis, K. & Wong, S. 2013. Risk reduction treatment of high-risk psychopathic offenders: the relationship of psychopathy and treatment change to violent recidivism. Personality Disorders, 4, 160-167.

Penney, S., Marshall, L. & Simpson, A. 2016. The assessment of dynamic risk among forensic psychiatric patients transitioning to the community. Law and Human Behavior, 40, 374-386.

Persson, M., Belfrage, H., Fredriksson, B. & Kristiansson, M. 2017. Violence during imprisonment, forensic psychiatric care, and probation: Correlations and predictive validity of the risk assessment instruments COVR, LSI-R, HCR-20(V3), and SAPROF. International Journal of Forensic Mental Health, 16, 117-129.

Pillay, S., Oliver, B., Butler, L. & Kennedy, H. 2008. Risk stratification and the care pathway. Irish Journal of Psychological Medicine, 25, 123-127.

Puzzo, I., Sedgwick, O., Kelly, R., Greer, B., Kumari, V., Guðjónsson, G. & Young, S. 2019. Attention problems predict risk of violence and rehabilitative engagement in mentally disordered offenders. Frontiers in Psychiatry, 10, 279.

Quinn, R., Miles, H. & Kinane, C. 2013.The validity of the Short-Term Assessment of Risk and Treatability (START) in a UK medium secure forensic mental health service. International Journal of Forensic Mental Health, 12, 215-224.

Ribeiro, R., Tully, J. & Fotiadou, M. 2015. Clinical characteristics and outcomes on discharge of women admitted to a Medium Secure Unit over a 4-year period. International Journal of Law and Psychiatry, 39, 83-89.

Richter, M., O'Reilly, K., O'Sullivan, D., O'Flynn, P., Corvin, A., Donohoe, G., Coyle, C., Davoren, M., Higgins, C., Byrne, O., Nutley, T., Nulty, A., Sharma, K., O’Connell, P. & Kennedy, H. 2018. Prospective observational cohort study of 'treatment as usual' over four years for patients with schizophrenia in a national forensic hospital. BMC Psychiatry, 18, 289.

Romeva, G., Rubio, L., Guerre, S., Miravet, M., Caceres, A. & Thomas, S. 2010. Clinical validation of the CANFOR scale (Camberwell Assessment of Need-Forensic version) for the needs assessment of people with mental health problems in the forensic services. Actas Espanoles de Psiquiatria, 38, 129-137.

Ross, T., Reed, V., Fontao, M. & Pfaefflin, F. 2012. Assessing reliability, validity, and clinical utility of the BEST-Index in measuring living skills among forensic inpatients. International Journal of Offender Therapy and Comparative Criminology, 56, 385-400.

Ross, T., Woods, P., Reed, V., Sookoo, S., Dean, A., Kettles, A., Almvik, R., ter Horst, P., Brown, I., Collins, M., Walker, H. & Pfaefflin, F. 2008. Assessing living skills in forensic mental health care with the behavioural status index: A European network study. Psychotherapy Research, 18, 334-344.

Ross, T., Woods, P., Reed, V., Sookoo, S., Dean, A., Kettles, A., Almvik, R., ter Horst, P., Collins, M., Walker, H. & Pfaefflin, F. 2007. Selecting and monitoring living skills in forensic mental health care: Cross-border validation of the BEST-Index. International Journal of Mental Health, 36, 3-16.

Segal, A., Daffern, M., Thomas, S. & Ferguson, M. 2010. Needs and risks of patients in a state-wide inpatient forensic mental health population. International Journal of Mental Health Nursing, 19, 223-230.

Shinkfield, G. & Ogloff, J. 2016. Comparison of HoNOS and HoNOS-Secure in a forensic mental health hospital. Journal of Forensic Psychiatry and Psychology, 27, 867-885.

Sugarman, P., Walker, L. & Dickens, G. 2009. Managing outcome performance in mental health using HoNOS: experience at St Andrew's Healthcare. Psychiatric Bulletin, 33, 285-288.

Talina, M., Thomas, S., Cardoso, A., Aguiar, P., Caldas de Almeida, J. & Xavier, M. 2013. CANFOR Portuguese version: Validation study. BMC Psychiatry, 13, 157.

Thomas, S., Slade, M., McCrone, P., Harty, M., Parrott, J., Thornicroft, G. & Leese, M. 2008. The reliability and validity of the forensic Camberwell Assessment of Need (CANFOR): a needs assessment for forensic mental health service users. International Journal of Methods in Psychiatric Research, 17, 111-120.

Trizna, M. & Adamowski, T. 2016. Assessment of needs and clinical parameters in forensic patients in low and medium security wards. Archives of Psychiatry and Psychotherapy, 18, 48-57.

Troquete, N., Brink, R., Beintema, H., Mulder, T., Os, T., Schoevers, R. & Wiersma, D. 2015. Predictive validity of the short-term assessment of risk and treatability for violent behavior in outpatient forensic psychiatric patients. Psychological Assessment, 27, 377-391.

Tully, J., Cappai, A., Lally, J. & Fotiadou M. 2019. Follow-up study of 6.5 years of admissions to a UK female medium secure forensic psychiatry unit. BJPsych Bulletin, 43, 54-57.

Viljoen, S., Nicholls, T., Greaves, C., de Ruiter C. & Brink, J. 2011. Resilience and successful community reintegration among female forensic psychiatric patients: A preliminary investigation. Behavioral Sciences and the Law, 29, 752-770.

Walker, H., Tulloch, L., Boa, K. & Ritchie, G. 2019. A multi-site survey of forensic nursing assessment. Journal of Forensic Practice, 21, 124-138.

Whittington, R., Bjorngaard, J., Brown, A., Nathan, R., Noblett, S. & Quinn, B. 2014. Dynamic relationship between multiple START assessments and violent incidents over time: a prospective cohort study. BMC Psychiatry, 14, 323.

Wilson, C., Desmarais, S., Nicholls, T. & Brink, J. 2010. The role of client strengths in assessments of violence risk using the Short- Term Assessment of Risk and Treatability (START). International Journal of Forensic Mental Health, 9, 282-293.

Wilson, C., Desmarais, S., Nicholls, T., Hart, S. & Brink, J. 2013. Predictive validity of dynamic factors: assessing violence risk in forensic psychiatric inpatients. Law and Human Behavior, 37, 377-388.

Wilson, K., Freestone, M., Taylor, C., Blazey, F. & Hardman, F. 2014. Effectiveness of modified therapeutic community treatment within a medium-secure service for personality-disordered offenders. Journal of Forensic Psychiatry and Psychology, 25, 243-261.

Wong, S. & Gordon, A. 2006. The validity and reliability of the Violence Risk Scale: A treatment-friendly violence risk assessment tool. Psychology Public Policy and Law, 12, 279-309.

Woods, P. 1999. The Behavioural Status Index (BSI): Descriptive Studies within a Forensic Context. PHD. Thesis, Anglia Polytechnic University, Cambridge.

Woods, P. & Reed, V. 1999. The Behavioural Status Index (BSI) some preliminary reliability studies. International Journal of Psychiatric Nursing Research, 5, 554-561.

Woods, P., Reed, V. & Robinson, D. 1999. The Behavioural Status Index: therapeutic assessment of risk, insight, communication and social skills. Journal of Psychiatric and Mental Health Nursing, 6, 79-90.

Woods, P., Reed, V. & Collins, M. 2001. Measuring communication and social skills in a high security forensic setting using the behavioural status index. International Journal of Psychiatric Nursing Research, 7, 761-777.

Woods, P., Reed, V. & Collins M. 2001a. Measuring insight in a high‐security forensic setting using the Behavioural Status Index. British Journal of Forensic Practice, 3, 3-12.

Woods, P., Reed, V. & Collins M. 2001b. Measuring risk in a high security forensic setting through the behavioural status index. International Journal of Psychiatric Nursing Research, 7, 793-805.

Woods, P., Reed, V. & Collins M. 2003a. Exploring core relationships between insight and communication and social skills in mentally disordered offenders. Journal of Psychiatric and Mental Health Nursing, 10, 518-525.

Woods, P., Reed, V. & Collins, M. 2003b. The relationship between risk and insight in a high-security forensic setting. Journal of Psychiatric and Mental Health Nursing, 10, 510-507.

Woods, P., Reed, V. & Collins, M. 2004. Relationships among risk, and communication and social skills in a high security forensic setting. Issues in Mental Health Nursing, 25, 769-782.

Woods, P., Reed, V. & Collins, M. 2005. The Behavioural Status Index: testing a social risk assessment model in a high security forensic setting. Journal of Forensic Nursing, 1, 9-19.

Yoon, D., Spehr, A. & Briken, P. 2011. Structured assessment of protective factors: a German pilot study in sex offenders. Journal of Forensic Psychiatry and Psychology. 22, 834-844.
