# Supplementary material for: Outcome measures in forensic mental health services: A systematic review of instruments and qualitative evidence synthesis
Source: Eur Psychiatry. 2021 May 28;64(1):e37. doi: 10.1192/j.eurpsy.2021.32 (PMC8260563; doi:10.1192/j.eurpsy.2021.32)
Supplement: Supplementary file 1 [file S0924933821000328sup001.zip › S0924933821000328sup005.docx]

**Table A4**

*Summary of evidence for psychometric properties of instruments identified in the systematic review*

| Instrument | Instrument development and content validity | Other psychometric properties |
| --- | --- | --- |
| Historical, Clinical, Risk – 20 (HCR-20) Version 3 | The Historical, Clinical, Risk – 20 (HCR-20) Version 3 is the latest of the well-established HCR tools, following on from version 1 (1995) and version 2 (1997) [1]. The authors developed a draft of the third version of the HCR-20 in 2008, in order to incorporate the latest research since the previous versions and improve its clinical utility. This combined literature reviews, feedback at conferences, beta testing with small groups of clinicians on three sites in different countries, followed by piloting in six different countries. It was not clear if the literature reviews were systematic or otherwise structured. There is some limited evidence that users find version 3 an improvement over previous versions, but no structured qualitative evidence for the content validity of HCR 20 as an outcome measure was identified [2, 3]. | A total of 18 studies contained relevant evidence for other psychometric properties, including 2340 participants (see Table A3, Supplementary Material 4). No studies were identified that consider the structural validity of the HCR-20. Evidence for internal consistency was derived from 4 studies, at least 3 of which were of good quality [2, 4-6]. The values of Cronbach α were very variable, ranging from 0.33 to 0.87. Reliability was examined in 10 studies, including 8 of adequate quality [5, 7-15]. Only interrater reliability was considered. Despite some variability, overall the intra-class correlation (ICC) scores were adequate (0.76-0.94 for the total HCR-20 score). Testing hypotheses for construct validity was carried out in all but 1 study [5], including the difference between subgroups, such as level of security and sex, correlation with other measures, such as the START, and prediction of a range of outcomes, including violence. A total of 682 relevant hypotheses were identified, of which 341 (50%) were supported. Responsiveness was studied in 3 papers, all of good quality [5, 13, 16]. Clinical and risk scales showed evidence of change in 2 out of the 3 studies [13, 16]. |
| Short Term Assessment of Risk and Treatability (START) | The Short Term Assessment of Risk and Treatability (START) was developed by a team based in Canada, with overlaps with the authorship of the HCR-20. It was based on an earlier tool called the Short-Term Assessment of Risk (STAR) [17]. The development of the STAR and subsequent versions of the START involved self-reflection by the authors, unstructured consultation with colleagues to select items and reviews of the literature [18]. A study of 12 staff members in a medium secure unit in the UK found that several participants felt START was useful to organise information about a patient, but raised concerns about uncertainty of the timeframe and the subjectivity of assessments [19]. A systematic review of the START identified seven studies that considered feasibility and utility [20]. | A total of 28 studies were identified, which contained information on the START’s other psychometric properties (see Table A3, Supplementary Material 4). These studies contained up to 4740 participants, although it was unclear in one study if the number referred to participants or assessments [21]. One study used an earlier version of the START, which contained the same items as later versions, but was rated on a continuous six point scale from a very high risk to very high strength, rather than two separate scales for strengths and vulnerabilities [22]. There were no studies that explored the structural validity of the START. There were 5 high quality studies that considered internal consistency, with Cronbach’s α score consistently >0.70, indicating a high consistency [4, 22-25]. A total of 10 studies were identified that contained information about reliability [4, 16, 24-31]. Most focused on inter-rater reliability, but 1 did consider test-retest reliability [29]. There was considerable variability in both the quality and results of the studies, with ICC values ranging from 0.30-0.95. All 28 studies testing hypotheses for construct validity, including difference between subgroups, such as level of security and sex, correlation with other measures, such as HoNOS-Secure and HCR-20, and prediction of a range of outcomes, including violence, self-harm and victimisation. The results were highly variable between studies. A total of 1256 hypotheses for construct validity were tested, with 601 (48%) being supported. Only 2 studies considered responsiveness, with one of poor quality [23]. The second study was of good quality and concluded that the START showed adequate responsiveness [16]. |
| Camberwell Assessment of Need – Forensic Version (CANFOR) | The Camberwell Assessment of Need – Forensic Version (CANFOR) is part of a family of needs assessment instruments designed to be used with different populations of mental health service users. The original version, the Camberwell Assessment of Need (CAN) was developed in the 1990s by a team at the Institute of Psychiatry at King’s College London [32]. The CAN was used as a template to develop the CANFOR [33]. The authors reworded original items and additional domains were added. This process was carried out by a team of five professionals from different disciplines. This draft was then piloted with 20 service users and 17 staff members and revisions were made based on the feedback obtained. Content validity was then investigated by interviewing 60 services users, who were asked to rate the relevance of each item on a four point scale. Two additional items were suggested, but these referred to intervention, not needs, so were not further considered. Fifty professionals were also surveyed to ascertain their views on the need for the CANFOR, the relevance of the ratings, the length of the scale and its comprehensiveness. Comprehensibility was investigated through the application of the Flesch ease of reading scale, on which the CANFOR scored 59, indicating that it was at the appropriate level for most readers. | A total of 10 studies were identified considering other psychometric properties, involving 794 patient participants (see Table A3, Supplementary Material 4). Reliability was investigated in four studies, which all examined both inter-rater and test retest reliabilities [33-36]. Overall the methods appear appropriate and the values of ICCs and Cohen’s κ indicate adequate reliability, at least at the level of aggregate scale scores. Testing hypotheses for construct validity occurs in all studies and covers difference between subgroups, such as patients in decreasingly secure settings and correlation with other measures, such as the Global Assessment of Functioning (GAF) and Brief Psychiatric Rating Scale (BPRS). There were no studies that look at the predictive ability of CANFOR. In total 59 out of 96 (61%) hypotheses were supported. |
| Dangerousness Understanding, Recovery and Urgency Manual (DUNDRUM) | The Dangerousness Understanding, Recovery and Urgency Manual (DUNDRUM) is a linked toolkit of rating scales designed to be used at different stages of the forensic mental health pathway [37]. DUNDRUM 3 and 4 are designed to guide moves along the recovery pathway, to make decisions about readiness for a move to less secure inpatient setting or discharge to the community. Patient reported versions of DUNDRUM 3 and 4 were subsequently developed, which mirror the items in the original clinician version [38]. The DUNDRUM quartet was developed by a team at the Central Mental Hospital in the Republic of Ireland. The items in the scale are reportedly drafted based on a number of previously developed assessment criteria, decision algorithms and structured professional judgements along with the authors’ own experience and research. The DUNDRUM 3 and 4 are also based on existing scales such as the HCR-20, CANFOR and HoNOS and a number of theoretical models, including engagement, recovery, Maslow’s hierarchy of need, and the trans-theoretical stages of change [39, 40]. The authors explain that professional colleagues contributed to the DUNDRUM manual through comments, criticisms and feedback, although this process is not described in further detail. The content of the patient reported scales of the DUNDRUM 3 and 4 are described as being developed in consultation with one service user, in order to allow ease of interpretation, while ensuring fidelity to the clinician rated items [38]. The process of this consultation is not described in detail. The self-rated scales have been published in a later edition of the DUNDRUM manual [41]. | In total 8 studies were identified, involving 967 participants (see Table A3, Supplementary Material 4). Structural validity was examined in 1 study through a principal components factor analysis [42]. The results support the unidimensionality of the DUNDRUM 3 and 4 scales. No confirmatory factor analysis was performed and the analysis of 95 participants is less than the minimum sample size of 100 recommended by COSMIN [43]. Internal consistency is examined in four studies and the evidence demonstrates high values of Cronbach’s α for both the clinician and patient rated versions of DUNDRUM 3 and 4. Evidence for the reliability of the DUNDRUM 3 and 4 is limited, with only 1 study reporting values for inter-rater reliability [42]. There is no evidence of test-retest reliability. For the DUNDRUM 3, only 1 study of measurement error was identified, which calculated a reliable change index using statistical methods [44]. This study compared this to a measure of clinically meaningful change, which was based on the theoretical basis of the instrument linked to levels of security, rather than empirical qualitative methods. There was extensive testing of hypotheses for construct validity in all but one study [44], including the difference between subgroups such as those with leave and those without leave, correlation with a range of other instruments, such as the HCR-20, CANFOR and SAPROF and prediction of violence, self-harm and moves within the patient pathway. In total 145 out of 192 (76%) of hypotheses identified were supported. There is one study of responsiveness, which considers the DUNDRUM 3 scale only [44]. Overall the total sample shows change, however there is a mixed picture when this is broken down into two subgroups of longer and shorter stay patients. The proportion of patients showing change greater that the reliable change index (RCI) and clinically significant change are also calculated. No evidence for the responsiveness of the DUNDRUM 4 or the two patient self-reported scale was identified. |
| Health of the Nation Outcomes Scales – Secure (HoNOS-Secure) | The Health of the Nation Outcome Scales –Secure is part of the HoNOS family of outcome measurement instruments. The authors of the original HoNOS tested and modified it during a four-stage process, considering acceptability, structure and sensitivity to change [45]. The first version of the HoNOS for forensic services was developed in 2002 within a group of independent hospitals, now called St. Andrew’s Healthcare. This was initially known at the Mentally Disordered Offender (MDO) scale. It constituted an additional 7 items, which formed a security scale, rated alongside modified versions of the original 12 items from the original HoNOS [46]. This new scale was reported to correlate highly with the original HoNOS in an unpublished study in 5 secure units [47]. The second version was subsequently developed using ‘qualitative consultation and case vignettes in order to establish face and consensual validity’ and was published in 2004 [48]. | A total of 21 studies were identified involving 2440 participants (see Table A3, Supplementary Material 4). Two studies examined the internal consistency of the two HoNOS subscales [48, 49]. The values of Cronbach’s α suggest good internal validity, however given the absence of evidence for structural validity, these results were assumed to be of indeterminate quality [43]. Only one study examined the interrater reliability of the HoNOS Secure [48]. Despite being of adequate methodological quality, the study only examined individual items and not the reliability for the total or subscale scores. The values of the ICC were highly variable, with some items demonstrating good reliability and other poor reliability. Only one study considers measurement error and this is calculated by statistical methods [50]. Testing of hypotheses for construct validity was undertaken in 14 studies and includes prediction of violence, difference between subgroups such as gender, security level and legal status and correlation with other measures, including risk assessments, neuropsychological measures and measures of social functioning [47, 49, 51-62]. Overall 103 out of 268 hypotheses (38%) tested are substantiated. Prediction was only tested in one of these studies, but the AUCs suggest good predictive abilities, with the AUC for interpersonal violence at 1, 3 and 6 months 0.89, 0.78 and 0.78 [61]. Responsiveness was examined in 11 studies [47, 49, 50, 54, 62-68]. The resultant picture was mixed, with a total of 58 out of 120 pairings (48%) showing significant change. This was supported by the mixture of improvement and deterioration observed in the two studies considering clinically important change [49, 50]. |
| Level of Service: Case Management Inventory (LS/CMI) | The Level of Service: Case Management Inventory is the most recent iteration of a series of tools in the Level of Service Inventory family. The LS/CMI is an evolution of earlier tools, such as the Level of Service Inventory-Revised and incorporates many elements of its predecessors [69]. The LS/CMI is not explicitly developed for use in healthcare settings, such as forensic mental health hospitals or community services. Mental health is only considered in section 4 ‘other client issues’, which does not contribute to the overall score, but can be used for administrative override purposes and to inform case management [70]. There is no evidence for the involvement of relevant stakeholders from forensic mental health services in the development of the LS/CMI. | There was limited published evidence identified for the use of the LS/CMI in a forensic mental health setting. In total only three studies were identified and all of these only considered the general scales in section 1 (see Table A3, Supplementary Material 4). Two of the studies were set in an outpatient psychiatric facility involving participants who were offenders referred for psychiatric assessment [71, 72]. Evidence was only available for internal consistency and testing hypotheses for construct validity. Internal consistency was only considered in one study and only 6 out of the 8 subscales of the general risk/needs scale were calculated101 [71]. Internal consistency varied considerably between subscales from 0.80 for Criminal History to 0.07 for Antisocial Orientation. Testing hypotheses for construct validity was conducted in all three included studies, but was limited to correlations with other measures, including the CANFOR, HoNOS Secure and HCR-20. Out of 193 [55] hypotheses tested, 63 (35%) were supported with significant correlations. |
| Violence Risk Scale (VRS) | The Violence Risk Scale (VRS) was designed to be a generic risk assessment for ‘forensic clients’, in particular those that were ‘being considered for release from institutions to the community after a period of treatment’ [73]. The VRS is explicitly designed to measure change in an individual’s risk of violence over time, and therefore to provide an assessment of the effectiveness of treatment in reducing this risk. The VRS is based on the Transtheoretical Model of Change [40] and the psychology of criminal conduct and the principles of effective correctional treatment [74]. No specific evidence for content validity as an outcome measure was identified. | There were 13 studies identified containing evidence on other psychometric properties, involving at least 1852 participants (see Table A3, Supplementary Material 4). There appeared to be at least some overlap in the samples of two of the studies [75, 76]. Evidence for structural validity is limited to exploratory factor analyses performed by the tool’s authors [74]. The results suggest that the static variables lack unidimensionality, which may account for the internal consistency of these variables. There was no confirmatory factor analysis identified. The same study is the only evidence of internal consistency, which appears to be good for the total and dynamic scores. Evidence of good interrater reliability is directly available from 6 studies [16, 75, 77-80]. It is also reported from a previously unpublished source by Wong and Gordon [74]. Four studies consider the measurement error of the instrument [79, 81-83]. Only one uses a clinical, rather than just statistical, methodology [82] and none meet COSMIN criteria for adequate evidence [84]. Hypotheses for construct validity are tested in 11 different studies, including difference between subgroups, prediction of outcomes and correlation with other measures [16, 74-78, 80, 83, 85-87]. In total 223 out of 334 (67 %) hypotheses were supported. There was evidence regarding responsiveness from 8 studies, which give a mixed picture [16, 75, 76, 79-83]. Some suggested statistically significant change [16, 80, 81], but those considering change indices found either limited or no improvement [79, 82]. One study did show significant change that was also reliable for a majority of participants [83]. |
| Structured Assessment of Protective Factors for risk of violence (SAPROF) | The SAPROF was first published in Dutch in 2007 [88]. It was subsequently translated in to English and several other languages. The included items were based on literature reviews of protective factors and contextual factors. Clinical input consisted of asking a range of mental health professionals who participated in 60 case conferences to suggest factors that may be protective against a relapse in to violent behaviour. Items in the prototype version were reduced by field testing [89]. | Twelve studies were identified that contained information about the psychometric properties of SAPROF in a forensic psychiatric context (see Table A3, Supplementary Material 4). There was no evidence to support good structural validity and the three subscales did not appear to have been empirically validated. There are two studies of adequate quality that indicate good internal consistency for the overall scale [4, 90]. There is evidence from 8 studies for inter-rater reliability [4, 9, 10, 12, 90-93] . Despite some inconsistency, overall there is strong evidence for adequate reliability, especially of the total score. No studies were identified that examined test-retest reliability. Testing hypotheses for construct validity was conducted in all of the included studies, including predictive validity (mainly for violence), difference between subgroups and correlation with a number of other measures, particularly the HCR-20. In total 199 out of 321 (62%) results were in line with the hypotheses. Responsiveness was considered by two studies which compared pre and post treatment scores over a variable period of follow up [91, 93]. In total 14 out of 14 (100%) scores showed significant change after treatment. |
| Sexual Violence Risk 20 (SVR-20) | The SVR-20 is developed based on risk factors for sexual violence [94]. No specific evidence to support its content validity as an outcome measure was identified. | Five studies were identified containing information on other psychometric properties that involved forensic psychiatric assessment (see Table A3, Supplementary Material 4). There was some evidence for interrater reliability, which was acceptable for individual subscales and total score, but inadequate for the overall risk rating [10, 95]. Testing hypotheses for construct validity included differences between subgroups, correlation with other risk assessment tools and predictive abilities relative to a range of violent and sexually violent outcomes [10, 95-98] with 32 out of 74 (43%) hypotheses supported. |
| Behavioural Status Index (BEST) | The BEST Index is a clinician rated instrument, which was designed to provide an assessment of behaviour. Evidence is available about the process of its development and content validity from a number of sources. There is limited information about the numbers of participants and the exact methods of data collection or analysis [99-101]. | Evidence for other psychometric properties was identified in 4 studies, although some of these were reported in multiple papers (see Table A3, Supplementary Material 4). Evidence for structural validity provides a mixed picture and is limited to only three of the full six subscales, with confirmatory factor analysis of a three factor model identifying an inadequate level of fit [102, 103]. Good internal consistency is well supported by high Cronbach’s alpha scores, but there is some question about how to interpret these in light of the evidence of inadequate structural validity [103-105]. Reliability results are variable, with many scores suggestive of inadequate agreement [104-106]. Testing hypotheses for construct validity occurred in all 4 studies, including differences between subgroups, such as level of security, and correlation with other measures, such as the HCR-20. 202 out of 319 (63%) hypotheses are supported. The evidence for responsiveness from two studies is equivocal, with some subscales demonstrating significant change, although these subscales are not consistent between studies [104, 105]. |

Notes

Other psychometric properties include structural validity, internal consistency, measurement invariance, reliability, measurement error, hypothesis testing and responsiveness.

References

1. Douglas K, Hart S, Webster C, Belfrage H. HCR-20 V3 Assessing risk for violence: User guide. Burnaby, BC, Canada: Mental Health, Law, and Policy Institute, Simon Fraser University; 2013.

2. Bjorkly S, Eidhammer G, Selmer LE. Concurrent validity and clinical utility of the HCR-20V3 compared with the HCR-20 in forensic mental health nursing: similar tools but improved method. Journal of Forensic Nursing. 2014;10:234-42.

3. de Vogel V, van den Broek E, de Vries Robbe M. The use of the HCR-20V3 in Dutch forensic psychiatric practice. The International Journal of Forensic Mental Health. 2014;13(2):109-21.

4. Abidin Z, Davoren M, Naughton L, Gibbons O, Nulty A, Kennedy HG. Susceptibility (risk and protective) factors for in-patient violence and self-harm: Prospective study of structured professional judgement instruments START and SAPROF, DUNDRUM-3 and DUNDRUM-4 in forensic mental health services. BMC Psychiatry. 2013;13: 197.

5. Penney SR, Marshall LA, Simpson AI. The assessment of dynamic risk among forensic psychiatric patients transitioning to the community. Law & Human Behavior. 2016;40(4):374-86.

6. Adams J, Thomas SD, Mackinnon T, Eggleton D. The risks, needs and stages of recovery of a complete forensic patient cohort in an Australian state. BMC Psychiatry. 2018;18: 35.

7. Douglas KS, Belfrage H. Interrater Reliability and Concurrent Validity of the HCR-20 Version 3. International Journal of Forensic Mental Health. 2014;13:130-9.

8. Doyle M, Power LA, Coid J, Kallis C, Ullrich S, Shaw J. Predicting post-discharge community violence in England and Wales using the HCR-20 (V3). International Journal of Forensic Mental Health. 2014;13:140-7.

9. Coid JW, Kallis C, Doyle M, Shaw J, Ullrich S. Identifying causal risk factors for violence among discharged patients. PLoS ONE. 2015;10:e0142493.

10. de Vries Robbe M, de Vogel V, Koster K, Bogaerts S. Assessing protective factors for sexually violent offending with the SAPROF. Sexual Abuse: Journal of Research & Treatment. 2015;27:51-70.

11. Green D, Schneider M, Griswold H, Belfi B, Herrera M, DeBlasi A. A comparison of the HCR-20(V3) among male and female insanity acquittees: a retrospective file study. International Journal of Forensic Mental Health. 2016;15:48-64.

12. Persson M, Belfrage H, Fredriksson B, Kristiansson M. Violence during imprisonment, forensic psychiatric care, and probation: correlations and predictive validity of the risk assessment instruments COVR, LSI-R, HCR-20(V3), and SAPROF. International Journal of Forensic Mental Health. 2017;16:117-29.

13. Mastromanno B, Brookstein DM, Ogloff JR, Campbell R, Chu CM, Daffern M. Assessing change in dynamic risk factors in forensic psychiatric inpatients: Relationship with psychopathy and recidivism. Journal of Forensic Psychiatry & Psychology. 2018;29:323-36.

14. Grossi LM, Green D, Griswold H, Cabeldue M, Belfi B. Assessing inpatient victimization risk among insanity acquittees using the HCR-20(V3). Journal of the American Academy of Psychiatry and the Law. 2019;47:286-98.

15. Cabeldue M, Green D, Griswold H, Schneider M, Smith J, Belfi B, et al. Using the HCR-20(V3) to differentiate insanity acquittees based on opinions of readiness for transfer. Journal of the American Academy of Psychiatry and the Law. 2018;46:339-50.

16. Hogan NR, Olver ME. Assessing risk for aggression in forensic psychiatric inpatients: An examination of five measures. Law & Human Behavior. 2016;40:233-43.

17. Webster C, Nicholls T, Martin M, Desmarais S, Brink J. Short-Term Assessment of Risk and Treatability (START): The case for a new structured professional judgment scheme. Behavioral Sciences and the Law. 2006;24:747-66.

18. Webster C, Martin M, Brink J, Nicholls T, Desmarais S. Short-Term Assessment of Risk and Treatability (START). Port Coquitlam, BC, Canada: Forensic Psychiatric Services Commission, St. Joseph’s Healthcare; 2009.

19. Doyle M, Lewis G, Brisbane M. Implementing the Short-term Assessment of Risk and Treatability (START) in a forensic mental health service. Psychiatric Bulletin. 2008;32:406-8.

20. O'Shea LE, Dickens GL. Short-Term Assessment of Risk and Treatability (START): systematic review and meta-analysis. Psychological Assessment. 2014;26:990-1002.

21. Nicholls T, Petersen K, Brink J, Webster C. A clinical and risk profile of forensic psychiatric patients: Treatment team STARTs in a Canadian service. The International Journal of Forensic Mental Health. 2011;10:187-99.

22. Nicholls T, Brink J, Desmarais S, Webster C, Martin M. The Short-Term Assessment of Risk and Treatability (START): a prospective validation study in a forensic psychiatric dample. Assessment. 2006;13:313-27.

23. Nonstad K, Nesset MB, Kroppan E, Pedersen TW, Nottestad JA, Almvik R, et al. Predictive validity and other psychometric properties of the Short-Term Assessment of Risk and Treatability (START) in a Norwegian high secure hospital. The International Journal of Forensic Mental Health. 2010;9:294-9.

24. Viljoen S, Nicholls T, Greaves C, de Ruiter C, Brink J. Resilience and successful community reintegration among female forensic psychiatric patients: A preliminary investigation. Behavioral Sciences & the Law. 2011;29:752-70.

25. Lam J. Use of the Short Term Assessment of Risk and Treatability in a forensic facility: Examining the impact of suicide behavior on multiple risk outcomes. Dissertation Abstracts International: Section B: The Sciences and Engineering. 2015;76(3-B(E)).

26. Nicholls TL, Brink J, Desmarais SL, Webster CD, Martin M. The Short-Term Assessment of Risk and Treatability (START): a prospective validation study in a forensic psychiatric dample. Assessment. 2006;13:313-27.

27. Wilson C, Desmarais S, Nicholls T, Brink J. The role of client strengths in assessments of violence risk using the Short-Term Assessment of Risk and Treatability (START). The International Journal of Forensic Mental Health. 2010;9:282-93.

28. Desmarais SL, Nicholls TL, Wilson CM, Brink J. Using dynamic risk and protective factors to predict inpatient aggression: reliability and validity of START assessments. Psychological Assessment. 2012;24:685-700.

29. Whittington R, Bjorngaard JH, Brown A, Nathan R, Noblett S, Quinn B. Dynamic relationship between multiple START assessments and violent incidents over time: a prospective cohort study. BMC Psychiatry. 2014;14:323.

30. Troquete N, Brink R, Beintema H, Mulder T, Os T, Schoevers R, et al. Predictive validity of the Short-Term Assessment of Risk and Treatability for violent behavior in outpatient forensic psychiatric patients. Psychological assessment. 2015; 27:377-91.

31. O'Shea L, Dickens G. Role of assessment components and recent adverse outcomes in risk estimation and prediction: Use of the Short Term Assessment of Risk and Treatability (START) in an adult secure inpatient mental health service. Psychiatry Research. 2016;240:398-405.

32. Phelan M, Slade M, Thornicroft G, Dunn G, Holloway F, Wykes T, et al. The Camberwell Assessment of Need: the validity and reliability of an instrument to assess the needs of people with severe mental illness. British Journal of Psychiatry. 1995;167:589-95.

33. Thomas SD, Slade M, McCrone P, Harty MA, Parrott J, Thornicroft G, et al. The reliability and validity of the forensic Camberwell Assessment of Need (CANFOR): a needs assessment for forensic mental health service users. International Journal of Methods in Psychiatric Research. 2008;17:111-20.

34. Romeva GE, Rubio LG, Guerre SO, Miravet MJ, Caceres AG, Thomas SD. Clinical validation of the CANFOR scale (Camberwell Assessment of Need-Forensic version) for the needs assessment of people with mental health problems in the forensic services. Actas Espanolas de Psiquiatria. 2010;38:129-37.

35. Talina M, Thomas S, Cardoso A, Aguiar P, Caldas de Almeida JM, Xavier M. CANFOR Portuguese version: validation study. BMC Psychiatry. 2013;13:157.

36. Castelletti L, Lasalvia A, Molinari E, Thomas S, Stratico E, Bonetto C. A standardised tool for assessing needs in forensic psychiatric population: Clinical validation of the Italian CANFOR, staff version. Epidemiology and Psychiatric Sciences. 2015;24:274-81.

37. Kennedy H, O'Neill C, Flynn G, Gill P. Dangerousness Understanding, Recovery and Urgency Manual (The DUNDRUM quartet) Dublin, Ireland: Central Mental Hospital, National Forensic Mental Health Service and Academic Department of Psychiatry, University of Dublin, Trinity College; 2010.

38. Davoren M, Hennessy S, Conway C, Marrinan S, Gill P, Kennedy HG. Recovery and concordance in a secure forensic psychiatry hospital - the self rated DUNDRUM-3 programme completion and DUNDRUM-4 recovery scales. BMC Psychiatry. 2015;15:61.

39. Maslow AH. A theory of human motivation. Psychological Review. 1943;50:370-96.

40. Prochaska JO, DiClemente CC. Transtheoretical therapy: Toward a more integrative model of change. Psychotherapy: Theory, Research & Practice. 1982;19:276-88.

41. Kennedy H, O'Neill C, Flynn G, Gill P, Davoren M. Dangerousness Understanding, Recovery and Urgency Manual (The DUNDRUM quartet): Four Structured Professional Judgement Instruments for Admission Triage, Urgency, Treatment Completion and Recovery Assessments 2013.Version 1.0.26. Dublin, Ireland: Central Mental Hospital, National Forensic Mental Health Service and Academic Department of Psychiatry, University of Dublin, Trinity College; 2013.

42. O'Dwyer S, Davoren M, Abidin Z, Doyle E, McDonnell K, Kennedy HG. The DUNDRUM Quartet: validation of structured professional judgement instruments DUNDRUM-3 assessment of programme completion and DUNDRUM-4 assessment of recovery in forensic mental health services. BMC Research Notes. 2011;4:229.

43. Prinsen CAC, Mokkink LB, Bouter LM, Alonso J, Patrick DL, de Vet HCW, et al. COSMIN guideline for systematic reviews of patient-reported outcome measures. Quality of Life Research. 2018;27:1147-57.

44. Richter MS, O'Reilly K, O'Sullivan D, O'Flynn P, Corvin A, Donohoe G, et al. Prospective observational cohort study of 'treatment as usual' over four years for patients with schizophrenia in a national forensic hospital. BMC Psychiatry. 2018;18(1):289.

45. Wing JK, Beevor AS, Curtis RH, Park SGB, Hadden J, Burns A. Health of the Nation Outcome Scales (HoNOS): Research and development. British Journal of Psychiatry. 1998;172:11-8.

46. Royal College of Psychiatrists. Health of the Nation Outcome Scales (HoNOS). London, UK: Royal College of Psychiatrists, 2020.

47. Dickens G, Sugarman P, Picchioni M, Long C. HoNOS-Secure: Tracking risk and recovery for men in secure care. The British Journal of Forensic Practice. 2010;12:36-46.

48. Dickens G, Sugarman P, Walker L. HoNOS-secure: A reliable outcome measure for users of secure and forensic mental health services. Journal of Forensic Psychiatry & Psychology. 2007;18:507-14.

49. Dickens GL, O'Shea LE. Reliable and Clinically Significant Change in Outcomes for Forensic Mental Health Inpatients: Use of the HoNOS-Secure. International Journal of Forensic Mental Health. 2017;16:161-71.

50. Longdon L, Edworthy R, Resnick J, Byrne A, Clarke M, Cheung N, et al. Patient characteristics and outcome measurement in a low secure forensic hospital. Criminal Behaviour & Mental Health. 2018;28:255-69.

51. Murphy D. Theory of mind functioning in mentally disordered offenders detained in high security psychiatric care: its relationship to clinical outcome, need and risk. Criminal Behaviour & Mental Health. 2007;17:300-11.

52. Pillay SM, Oliver B, Butler L, Kennedy HG. Risk stratification and the care pathway. Irish Journal of Psychological Medicine. 2008;25:123-7.

53. Segal A, Daffern M, Thomas S, Ferguson M. Needs and risks of patients in a state-wide inpatient forensic mental health population. International Journal of Mental Health Nursing. 2010;19:223-30.

54. Long C, Dickens G, Sugarman P, Craig L, Mochty U, Hollin C. Tracking risk profiles and outcome in a medium secure service for women: Use of the HoNOS-Secure. The International Journal of Forensic Mental Health. 2010;9:215-25.

55. Long CG, Fulton B, Dolley O, Hollin CR. Social problem-solving interventions in medium secure s ettings for women. Medicine, Science and the Law. 2011;51:215-9.

56. Abou-Sinna R, Luebbers S. Validity of assessing people experiencing mental illness who have offended using the Camberwell Assessment of Need-Forensic and Health of the Nation Outcome Scales-Secure. International Journal of Mental Health Nursing. 2012;21:462-70.

57. Quinn R, Miles H, Kinane C. The validity of the Short-Term Assessment of Risk and Treatability (START) in a UK medium secure forensic mental health service. International Journal of Forensic Mental Health. 2013;12:215-24.

58. Baliousis M, Huband N, Duggan C, McCarthy L, Vollm B. Development and validation of a treatment progress scale for personality disordered offenders. Personality & Mental Health. 2015;9:107-23.

59. Fan C-W. Psychometric properties and descriptive characteristics of clients by using two theory-based assessments. Dissertation Abstracts International: Section B: The Sciences and Engineering. 2015;76(1-B(E)).

60. Shinkfield G, Ogloff J. Comparison of HoNOS and HoNOS-Secure in a forensic mental health hospital. Journal of Forensic Psychiatry & Psychology. 2016;27:867-85.

61. Finch B, Gilligan DG, Halpin SA, Valentine ME. The short- to medium-term predictive validity of static and dynamic risk-of-violence measures in medium- to low-secure forensic and civil inpatients. Psychiatry, Psychology and Law. 2017;24:410-27.

62. Griffiths C, Roychowdhury A, Girardi A. Seclusion: the association with diagnosis, gender, length of stay and HoNOS-secure in low and medium secure inpatient mental health service. Journal of Forensic Psychiatry and Psychology. 2018;29:1-18.

63. Sugarman P, Walker L, Dickens G. Managing outcome performance in mental health using HoNOS: experience at St Andrew's Healthcare. Psychiatric Bulletin. 2009;33:285-8.

64. Long C, Dolley O, Hollin C. Women in medium secure care: tracking treatment progress for changes in risk profiles and treatment engagement. Journal of Psychiatric and Mental Health Nursing. 2011;18:425-31.

65. Long C, Fulton B, Dolley O, Hollin C. Social problem-solving interventions in medium secure settings for women. Medicine, Science and the Law. 2011;51:215-9.

66. Ribeiro RB, Tully J, Fotiadou M. Clinical characteristics and outcomes on discharge of women admitted to a Medium Secure Unit over a 4-year period. International Journal of Law & Psychiatry. 2015;39:83-9.

67. Fox E, Krawczyk K, Staniford J, Dickens GL. A service evaluation of a 1-year dialectical behaviour therapy programme for women with borderline personality disorder in a low secure unit. Behavioural & Cognitive Psychotherapy. 2015;43:676-91.

68. Tully J, Cappai A, Lally J, Fotiadou M. Follow-up study of 6.5 years of admissions to a UK female medium secure forensic psychiatry unit. BJPsych Bulletin. 2019;43:54-7.

69. Andrews DA, Bonta J. The level of service inventory-revised: Toronto, ON, Canada: Multi-Health Systems; 2000.

70. Andrews DA, Bonta J, Wormith J. Level of Service/Case Management Inventory: An Offender Assessment System. Scoring Guide. Toronto, ON, Canada: Multi-Health Systems; 2004.

71. Jung S, Daniels MK, Friesen M, Ledi D. An examination of convergent constructs among Level of Service measures and other measures. Journal of Forensic Psychiatry & Psychology. 2012;23:601-19.

72. Jung S, Ledi D, Daniels MK. Evaluating the concurrent validity of the HCR-20 scales. Journal of Risk Research. 2013;16:697-711.

73. Wong S, Gordon A. Violence Risk Scale Manual: Saskatoon, SK, Canada: University of Saskatchewan; 2000.

74. Wong S, Gordon A. The validity and reliability of the Violence Risk Scale: A treatment-friendly violence risk assessment tool. Psychology Public Policy and Law. 2006;12:279-309.

75. Lewis K, Olver ME, Wong SC. The Violence Risk Scale: predictive validity and linking changes in risk with violent recidivism in a sample of high-risk offenders with psychopathic traits. Assessment. 2013;20:150-64.

76. Olver ME, Lewis K, Wong SCP. Risk reduction treatment of high-risk psychopathic offenders: the relationship of psychopathy and treatment change to violent recidivism. Personality disorders. 2013;4:160-7.

77. Grevatt M, Thomas-Peter B, Hughes G. Violence, mental disorder and risk assessment: Can structured clinical assessments predict the short-term risk of inpatient violence? Journal of Forensic Psychiatry & Psychology. 2004;15:278-92.

78. Dolan M, Fullam R. The validity of the Violence Risk Scale second edition (VRS-2) in a British forensic inpatient sample. Journal of Forensic Psychiatry & Psychology. 2007;18:381-93.

79. Draycott S, Kirkpatrick T, Askari R. An idiographic examination of patient progress in the treatment of dangerous and severe personality disorder: a reliable change index approach. Journal of Forensic Psychiatry & Psychology. 2012;23:108-24.

80. Coupland RBA, Olver ME. Assessing dynamic violence risk in a high-risk treated sample of violent offenders. Assessment. 2018;27:1886-1900.

81. Wilson K, Freestone M, Taylor C, Blazey F, Hardman F. Effectiveness of modified therapeutic community treatment within a medium-secure service for personality-disordered offenders. Journal of Forensic Psychiatry & Psychology. 2014;25:243-61.

82. Howden S, Midgley J, Hargate R. Violent offender treatment in a medium secure unit. Journal of Forensic Practice. 2018;20:102-11.

83. Horgan H, Charteris C, Ambrose D. The Violence Reduction Programme: An exploration of post-treatment risk reduction in a specialist medium-secure unit. Criminal Behaviour and Mental Health. 2019;29:286-95.

84. Mokkink LB, Prinsen CAC, Patrick DL, Alonso J, Bouter LM, de Vet HCW, et al. COSMIN methodology for systematic reviews of Patient‐Reported Outcome Measures (PROMs). Amsterdam, The Netherlands: VU University Medical Centre; 2018.

85. Dolan M, Fullam R, Logan C, Davies G. The Violence Risk Scale Second Edition (VRS-2) as a predictor of institutional violence in a British forensic inpatient sample. Psychiatry Research. 2008;158:55-65.

86. Langton CM, Hogue TE, Daffern M, Mannion A, Howells K. Prediction of institutional aggression among personality disordered forensic patients using actuarial and structured clinical risk assessment tools: prospective evaluation of the HCR-20, VRS, Static-99, and Risk Matrix 2000. Psychology Crime & Law. 2009;15:635-59.

87. Langton CM. Personality traits and dynamic variables associated with types of aggression in high security forensic psychiatric inpatients. Dissertation Abstracts International: Section B: The Sciences and Engineering. 2011;72(1-B):541.

88. de Vogel V, de Ruiter C, Bouman Y, M. de Vries Robbe. SAPROF. Richtlijnen voor het beoordelen van beschermende factoren voor gewelddadig gedrag. Versie 1. [SAPROF. Guidelines for the assessment of protective factors for violence risk. Version 1]. Utrecht, The Netherlands: Forum Educatief; 2007.

89. de Vogel V, de Ruiter C, Bouman Y, de Vries Robbe M. Structured Assessment of Protective Factors for risk of violence (SAPROF): Guidelines for the assessment of protective factors for violence risk. 2nd ed. Utrecht, The Netherlands: Forum Educatif; 2012.

90. Kashiwagi H, Kikuchi A, Koyama M, Saito D, Hirabayashi N. Strength-based assessment for future violence risk: a retrospective validation study of the Structured Assessment of PROtective Factors for violence risk (SAPROF) Japanese version in forensic psychiatric inpatients. Annals of General Psychiatry. 2018;17:5.

91. de Vries Robbe M, de Vogel V, de Spa E. Protective factors for violence risk in forensic psychiatric patients: A retrospective validation study of the SAPROF. The International Journal of Forensic Mental Health. 2011;10:178-86.

92. de Vries Robbe M, de Vogel V, Douglas KS. Risk factors and protective factors: A two-sided dynamic approach to violence risk assessment. Journal of Forensic Psychiatry & Psychology. 2013;24:440-57.

93. de Vries Robbe M, de Vogel V, Douglas KS, Nijman HL. Changes in dynamic risk and protective factors for violence during inpatient forensic psychiatric treatment: predicting reductions in postdischarge community recidivism. Law & Human Behavior. 2015;39:53-61.

94. Boer D. Manual for the Sexual Violence Risk-20: Professional guidelines for assessing risk of sexual violence: British Columbia Institute Against Family Violence; 1997.

95. de Vogel V, de Ruiter C, van Beek D, Mead G. Predictive validity of the SVR-20 and Static-99 in a Dutch sample of treated sex offenders. Law & Human Behavior. 2004;28:235-51.

96. Yoon D, Spehr A, Briken P. Structured assessment of protective factors: a German pilot study in sex offenders. Journal of Forensic Psychiatry & Psychology. 2011;22:834-44.

97. Craig LA, Browne KD, Stringer I. Comparing sex offender risk assessment measures on a UK sample. International Journal of Offender Therapy & Comparative Criminology. 2004;48:7-27.

98. Craig LA, Beech A, Browne KD. Cross-validation of the risk matrix 2000 sexual and violent scales. Journal of Interpersonal Violence. 2006;21:612-33.

99. Reed V, Woods P. The Behavioural Status Index (BEST-Index) - A 'life skills' assessment for selecting and monitoring therapy in mental health care. 2nd edition. London, UK: Psychometric Press; 2002.

100. Magoub NA. Bridging therapy in hospital and community based psychiatric nursing care: a comparative study: Sheffield, UK: Sheffield City Polytechnic; 1988.

101. Robinson D, Reed V, Lange A. Developing risk assessment scales in forensic psychiatric care. Psychiatric Care. 1996;3:146-52.

102. Chakhssi F, de Ruiter C, Bernstein D. Reliability and validity of the Dutch version of the Behavioural Status Index: a nurse-rated forensic assessment tool. Assessment. 2010;17:58-69.

103. Woods P, Reed V, Robinson D. The Behavioural Status Index: therapeutic assessment of risk, insight, communication and social skills. Journal of Psychiatric and Mental Health Nursing. 1999;6:79-90.

104. Walker H, Tulloch L, Boa K, Ritchie G. A multi-site survey of forensic nursing assessment. Journal of Forensic Practice. 2019;21:124-38.

105. Ross T, Woods P, Reed V, Sookoo S, Dean A, Kettles A, et al. Assessing living skills in forensic mental health care with the behavioural status index: A European network study. Psychotherapy Research. 2008;18:334-44.

106. Woods P, Reed V. The Behavioural Status Index (BSI) some preliminary reliability studies. International Journal of Psychiatric Nursing Research. 1999;5:554-61.
