# Supplementary material for: Outcome measures in forensic mental health services: A systematic review of instruments and qualitative evidence synthesis
Source: Eur Psychiatry. 2021 May 28;64(1):e37. doi: 10.1192/j.eurpsy.2021.32 (PMC8260563; doi:10.1192/j.eurpsy.2021.32)
Supplement: Supplementary file 1 [file S0924933821000328sup001.zip › S0924933821000328sup002.docx]

**Table A1**

*Type of paper, type of study and geographical origin of the full text articles identified by the literature search which were used to identify instruments used as outcome measures in forensic mental health services.*

| **Format of paper** | **N** |
| --- | --- |
| Journal article | 456 |
| Conference proceedings | 22 |
| Dissertation | 13 |
| Book chapter | 7 |
| Research report | 2 |
| Letter | 2 |
| **Total** | **502** |
| **Type of study** |  |
| Psychometric | 247 |
| Observational | 186 |
| Descriptive | 30 |
| Trial | 17 |
| Systematic Review | 14 |
| Survey | 8 |
| **Total** | **502** |
| **Geographical region** |  |
| UK & Ireland | 227 |
| Rest of Europe | 141 |
| USA | 47 |
| Canada | 39 |
| Australia & NZ | 30 |
| Middle East and Asia | 10 |
| Latin America | 4 |
| Africa | 1 |
| Multiple regions | 3 |
| **Total** | **502** |
