# Supplementary material for: Outcome measures in forensic mental health services: A systematic review of instruments and qualitative evidence synthesis
Source: Eur Psychiatry. 2021 May 28;64(1):e37. doi: 10.1192/j.eurpsy.2021.32 (PMC8260563; doi:10.1192/j.eurpsy.2021.32)
Supplement: Supplementary file 1 [file S0924933821000328sup001.zip › S0924933821000328sup003.docx]

| Rank | Tool | Frequency | Multidimensional | Forensic | Selected |
| --- | --- | --- | --- | --- | --- |
| 1 | **Historical Clinical Risk 20 (HCR 20)** | 196 | Yes | Yes | Yes |
| 2 | **Short Term Assessment of Risk and Treatability (START)** | 53 | Yes | Yes | Yes |
| 3 | **Camberwell Assessment of Need - Forensic (CANFOR)** | 48 | Yes | Yes | Yes |
| 4 | Brief Psychiatric Rating Scale (BPRS) | 45 | No | No | No |
| 5 | **Dangerousness, Understanding, Recovery and Urgency Manual (DUNDRUM)** | 37 | Yes | Yes | Yes |
| 6 | Positive and Negative Symptom Scale (PANSS) | 38 | No | No | No |
| 7 | Overt Aggression Scale (OAS) | 35 | No | No | No |
| 8= | **Health of the Nation Outcome Scale - Secure (HoNOS-Secure)** | 31 | Yes | Yes | Yes |
| 8= | Globalised Assessment of Functioning (GAF) | 31 | No | No | No |
| 10 | **Level of Service Inventory (LSI)** | 28 | Yes | Yes | Yes |
| 11 | **Violence Risk Scale (VRS)** | 19 | Yes | Yes | Yes |
| 12= | **Structured Assessment of Protective factors for risk of violence (SAPROF)** | 16 | Yes | Yes | Yes |
| 12= | **Sexual Violence Risk 20 (SVR 20)** | 16 | Yes | Yes | Yes |
| 14 | **Behavioural Status Index (BEST)** | 15 | Yes | Yes | Yes |

**Table A2**

*Frequency of instrument citation and comparison with the eligibility criteria for further quality assessment*
