# Supplementary material for: Outcome measures in forensic mental health services: A systematic review of instruments and qualitative evidence synthesis
Source: Eur Psychiatry. 2021 May 28;64(1):e37. doi: 10.1192/j.eurpsy.2021.32 (PMC8260563; doi:10.1192/j.eurpsy.2021.32)
Supplement: Supplementary file 1 [file S0924933821000328sup001.zip › S0924933821000328sup001.docx]

Sample search strategy

The following search strategy was used in Medline, EMBASE and PsychINFO. The two strings were combined during the searching process to identify relevant records:

1. (tool? or instrument? or assess* or question* or scale or score or inventory or survey or status or report* or profile or measur*) adj3 (outcome or disability or recovery or risk or function* or symptom or 'qualit* of life' or QoL or rehab* or clinic* or well?being)
2. ((secure or forensic) adj3 (unit? or ward? or hospital? or department? or service* or psych* or team)) or 'mentally disordered offender'
